# Heat induces multiomic and phenotypic stress propagation in zebrafish embryos

**DOI:** 10.1093/pnasnexus/pgad137

**Published:** 2023-05-23

**Authors:** Lauric Feugere, Adam Bates, Timothy Emagbetere, Emma Chapman, Linsey E Malcolm, Kathleen Bulmer, Jörg Hardege, Pedro Beltran-Alvarez, Katharina C Wollenberg Valero

**Affiliations:** Department of Biological and Marine Sciences, University of Hull, Kingston upon Hull HU6 7RX, UK; Department of Biological and Marine Sciences, University of Hull, Kingston upon Hull HU6 7RX, UK; Wellcome Sanger Institute, Hinxton CB10 1SA, UK; Department of Biological and Marine Sciences, University of Hull, Kingston upon Hull HU6 7RX, UK; Department of Biological and Marine Sciences, University of Hull, Kingston upon Hull HU6 7RX, UK; Biomedical Institute for Multimorbidities, Centre for Biomedicine, Hull York Medical School, University of Hull, Kingston upon Hull HU6 7RX, UK; Biomedical Institute for Multimorbidities, Centre for Biomedicine, Hull York Medical School, University of Hull, Kingston upon Hull HU6 7RX, UK; Department of Biological and Marine Sciences, University of Hull, Kingston upon Hull HU6 7RX, UK; Biomedical Institute for Multimorbidities, Centre for Biomedicine, Hull York Medical School, University of Hull, Kingston upon Hull HU6 7RX, UK; School of Biology and Environmental Science, University College Dublin, Belfield Dublin 4, Ireland

**Keywords:** stress cues, stress propagation, stress response, thermal stress, multiomics

## Abstract

Heat alters biology from molecular to ecological levels, but may also have unknown indirect effects. This includes the concept that animals exposed to abiotic stress can induce stress in naive receivers. Here, we provide a comprehensive picture of the molecular signatures of this process, by integrating multiomic and phenotypic data. In individual zebrafish embryos, repeated heat peaks elicited both a molecular response and a burst of accelerated growth followed by a growth slowdown in concert with reduced responses to novel stimuli. Metabolomes of the media of heat treated vs. untreated embryos revealed candidate stress metabolites including sulfur-containing compounds and lipids. These stress metabolites elicited transcriptomic changes in naive receivers related to immune response, extracellular signaling, glycosaminoglycan/keratan sulfate, and lipid metabolism. Consequently, non-heat-exposed receivers (exposed to stress metabolites only) experienced accelerated catch-up growth in concert with reduced swimming performance. The combination of heat and stress metabolites accelerated development the most, mediated by apelin signaling. Our results prove the concept of indirect heat-induced stress propagation toward naive receivers, inducing phenotypes comparable with those resulting from direct heat exposure, but utilizing distinct molecular pathways. Group-exposing a nonlaboratory zebrafish line, we independently confirm that the glycosaminoglycan biosynthesis-related gene *chs1* and the mucus glycoprotein gene *prg4a*, functionally connected to the candidate stress metabolite classes sugars and phosphocholine, are differentially expressed in receivers. This hints at the production of *Schreckstoff*-like cues in receivers, leading to further stress propagation within groups, which may have ecological and animal welfare implications for aquatic populations in a changing climate.

**Significance Statement**

Aquatic animals utilize chemicals to mediate adaptive behaviors. For instance, predated fish release chemical cues that elicit antipredatory responses in naive receivers. But whether abiotic factors such as heat likewise alter chemical communication has received little focus. Here, we uncover a yet untested dimension of chemical communication—heat-stressed donors can induce stress in naive receivers. We show that heat activates molecular stress responses, leading to the release of distinct stress metabolite classes into the medium. These stress metabolites alter the transcriptome of receivers, resulting in faster development and hypoactivity. Heat combined with stress metabolites had the largest effect, highlighting that abiotic stress, experienced both directly and indirectly, can alter chemical communication and affect embryonic development.

## Introduction

Temperature is the abiotic “master” (1) factor regulating the biology of ectotherms (2). Heat alters a wide range of responses in fish, from gene expression (3) to development (4) and to population dynamics (5). The type of stimulus also matters: single and repeated periods of heat can induce different gene expression patterns leading to distinct behavioral stress responses (6). Repeated thermal conditioning can impair the ability to restore homeostasis (7) and alter the response to subsequent heat stress by attenuating the corticosteroid (8) and heat shock (9, 10) pathways. Furthermore, there is a growing concern about the response of fish embryos to heat due to their narrower thermal tolerance (11, 12), marking early development as a vulnerable “bottleneck” stage (13) when it comes to thermal stress. Heat stress responses at the molecular and behavioral levels are well studied in aquatic species, but its effect on chemical communication is less studied. Previous studies have focused mostly on cues released upon biotic factors such as injury (14) or disturbance (15). We have recently proposed that abiotic stress such as heat or low pH likewise elicits the release of olfactory cues, which we termed “stress metabolites” (SM) (16, 17). We defined SM as chemicals released, intentionally or not, by an animal in response to an abiotic stressor and that propagate stress responses to others (16, 17). They could be metabolic products of the stress response mechanism or have other sources. Such cues could either directly signal the presence of a stressor to others or receivers could interpret them as the activation of stress systems in senders (18). We found SM to elicit similar phenotypic responses as heat stress itself in naive receivers, which are the characteristics of a positive feedback loop (16). To date, only a few studies have shown that animals communicate to each other upon abiotic stress, such as heat and low pH (17, 19).

Zebrafish (*Danio rerio*) is a commonly used experimental model species, including for behavioral assays (20) and “omics” approaches (21). Importantly, the thermal biology of zebrafish is conserved in laboratory conditions, which makes it a suitable model for investigating climate-related questions (22), such as the mechanistic basis of heat stress perception. Zebrafish embryos produce alarm cues (23) and innately react to extracts of crushed conspecific larvae with a decreased activity as early as 12–24 h post fertilization (hpf; 24). Altogether, this shows that fish, including zebrafish at early embryonic stages, can detect and discriminate stress chemical cues.

**Fig. 1.**
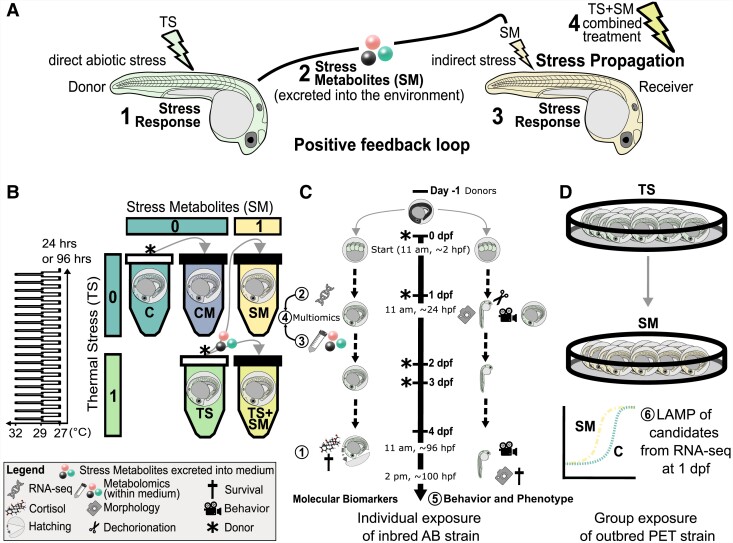
Scheme of experimental design. A) Hypotheses: abiotic stress causes (1) stress responses in aquatic animals, which (2) leads to the release of stress metabolites that (3) propagate stress responses to naive receivers. B) Zebrafish embryos (*D. rerio*) < 3 hpf were incubated according to a two-way factorial design represented by two predictors: thermal stress (0, control temperature of 27°C; 1, repeated thermal stress at a sublethal temperature of 32°C as shown in left graph) and SM (0, fresh medium free of metabolites; 1, SM released by embryos exposed to thermal stress). Treatments were CM, control metabolites at 27°C; C, control in fresh medium at 27°C; SM, stress metabolites at 27°C; TS, fresh medium in thermal stress; and TS + SM, stress metabolites in thermal stress. Arrows indicate medium transfer from metabolite donor (asterisk symbols) to metabolite receiver (plain black tube caps). C) Endpoints: individually exposed embryos were sampled for both molecular (pooled samples for RNA-seq and metabolomics of medium at 1 dpf and cortisol and HSP70 at 4 dpf) and phenotypic (1 dpf and 4 dpf) endpoints (circled numbers 1–5). D) Confirmatory experiment with LAMP data of candidate genes found in RNA-seq in group-exposed (20 embryos per petri dish) embryos incubated in stress metabolites (SM) or fresh medium (C) until 1 dpf. Endpoints 1/2/5 utilized laboratory inbred AB and endpoints 3/6 wilder PET embryos.

Natural chemical communication is likely mediated by a cocktail of compounds (25). However, stress-induced chemical communication has previously been studied using mainly phenotypic and physiological endpoints and using biotic disturbances in adult and juvenile stages (15). In contrast, work is still needed to unravel the molecular mechanisms and phenotypic consequences of stress propagation between social animals (18). To our knowledge, very few or no studies have utilized modern -omics methods to investigate the molecular basis of chemical stress propagation between sender and receiver in embryonic stages exposed to abiotic stress. Consequently, the biological compound bouquets mediating specifically heat stress-related communication, and their molecular pathways of action, remain unknown. Here, we explored the molecular response of zebrafish embryos exposed to thermal stress and to heat stress-induced metabolites using metabolomics and transcriptomics (Figs. 1 and S1A). We recently proposed (16, 17) a framework by which abiotic stress induces stress responses in aquatic animals (Hypothesis 1: also an initial postulate to verify before studying stress propagation), which release SM into their environment (Hypothesis 2) that propagate stress responses to naive receivers (Hypothesis 3), and that abiotic factors can alter the response to such SM (Hypothesis 4, Fig. 1A). We therefore expected that (i) the metabolomic profiles excreted by control and heat-stressed zebrafish embryos differ from each other. Since, to our knowledge, no prior study investigated the molecular pathways activated by stress-induced cues, we performed a transcriptome-wide analysis without a priori candidate pathways. However, we expected that (ii) there may be similarities between the transcriptomic signatures of embryos exposed to heat stress and to SM and (iii) that SM activate chemosensory perception genes and chemically responsive organs. (iv) Last, we expected both heat-stressed and indirectly stressed individuals to have distinct fitness consequences compared with controls (Fig. 1A). In the context of our study, we defined “stressors” as any factors that deviate homeostasis and “stress” as the state of threatened homeostasis, which can be assessed by a range of behavioral and physiological adaptive responses to restore homeostasis (26–29). In this contribution, we investigated the mechanistic basis underlying the end-to-end suite of events of stress propagation between directly abiotically stressed individuals and receivers of chemical SM (Fig. 1A).

## Results

In this study, we investigated the mechanistic basis of stress propagation between zebrafish embryos. We hypothesized that abiotically stressed individuals release SM into their environment that signal a risk to conspecifics, which alters their fitness-relevant responses through molecular pathways (Fig. 1A). We explored the different steps of our proposed stress propagation mechanism. (i) We measured molecular stress biomarkers and fitness-relevant phenotypes to confirm that heat-exposed embryos were stressed. (ii) We sequenced the transcriptome of heat-stressed embryos to identify the genes and functions leading to the release of SM. (iii) We used metabolomics to characterize the chemical nature of SM and their functional connections with heat-induced gene products. (iv) We sequenced the transcriptome of embryos incubated in SM to identify the genes and functions they alter in receivers and their connection with metabolite-induced gene products. (v) We investigated the whole-body consequences of stress propagation through phenotypic measurements to infer the biological relevance of this mechanism. (vi) We explored the effects of combined heat and SM treatments to infer the ecological relevance of stress propagation in a realistic setting where both abiotic stress and chemical cues cooccur.

### Heat stress, but not SM, leads to increased cortisol in 4-day post fertilization larvae

First, we aimed to confirm the preliminary condition that heat-exposed SM donors were stressed by the high-temperature protocol (Hypothesis 1, Fig. 1A) by measuring levels of two stress biomarkers, cortisol and HSP70. We first confirmed that cortisol increased between 1 and 4 days post fertilization (dpf) in control embryos (Fig. S2A and B). Cortisol was then measured at 4 dpf in unheated control (C) and heated (TS) medium donors and their respective receivers of media containing control metabolites (CM) or SM (Fig. S2C and Table S1). A one-way ANOVA showed that cortisol concentration significantly varied across treatments (*F* = 14.35, *P* = 0.0014). Post hoc pairwise comparisons revealed strong evidence that TS significantly increased cortisol concentrations by 92% compared with control C (*t* = −4.86, *P* = 0.0055, huge effect size = 6.17), but incubation media did not have an effect (Table S1). In contrast to cortisol, protein levels of HSP70 were similar across all treatments at 4 dpf (*F* = 1.9271, *P* = 0.2038, Fig. S2D).

### Heat stress alters the whole-body transcriptome of 1-dpf embryos

**Fig. 2.**
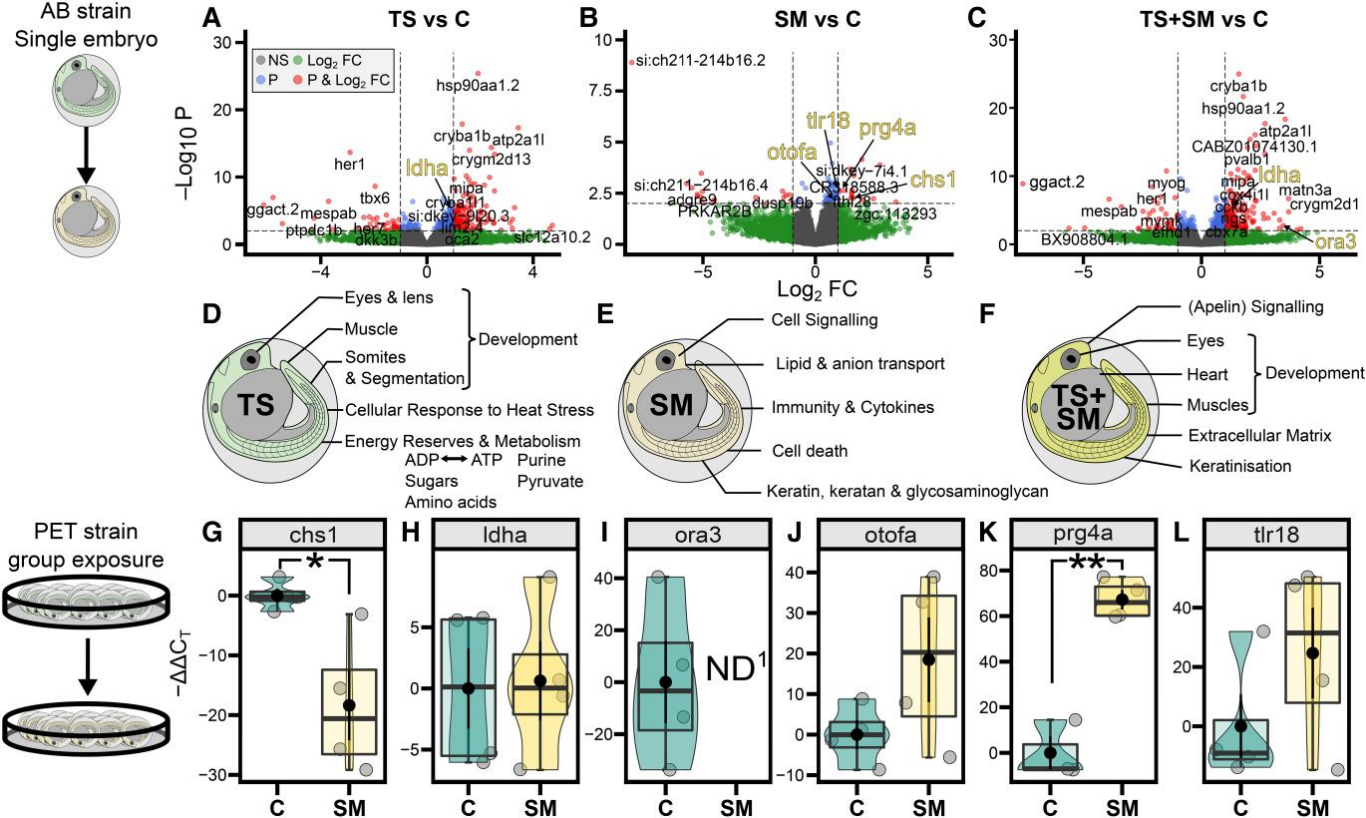
TS and SM alter the transcriptome and its functions in 1-dpf zebrafish embryos. Top row: volcano plots showing the DEGs in response to A) TS, B) SM, and C) their combination TS + SM compared with control C. Genes of interest are shown in red when they have significant raw *P* values (above horizontal line) and an absolute FC (representing the effect size) > 2 (|log 2 FC| > 1, vertical lines). DEGs left to the left vertical line and right to the right vertical line are respectively significantly underexpressed and overexpressed compared with the control C. Middle row: gene functional categories from KEGG, Reactome, and GO BP analysis for D) TS, E) SM, and F) those uniquely present in the combined treatment TS + SM. Top and middle rows show transcriptomic data from individually raised AB strain embryos. Bottom row shows LAMP data of candidate genes associated with stress metabolites showing the normalized time of detection (−ΔΔ*C*_T_) in C and SM in more realistic environmental conditions (genetically diversified outcrossing PET strain raised in groups). G) *chs1*, chitin synthase 1; H) *ldha*, lactate dehydrogenase A4; I) *ora3*, olfactory receptor class A related 3. ^1^ND, mRNA amplification nondetected suggesting a depletion of *ora3* in SM; J) *otofa*, otoferlin a; K) *prg4a*, proteoglycan 4a; L) *tlr18*, toll-like receptor 18. Student's *t* tests compared SM with C with significant comparisons shown by horizontal bars with *P ≤ 0.05 and **P ≤ 0.01. Treatments were SM, stress metabolites at 27°C; TS, fresh medium in thermal stress; and TS + SM, stress metabolites in thermal stress, compared with C, control in fresh medium at 27°C.

**Fig. 3.**
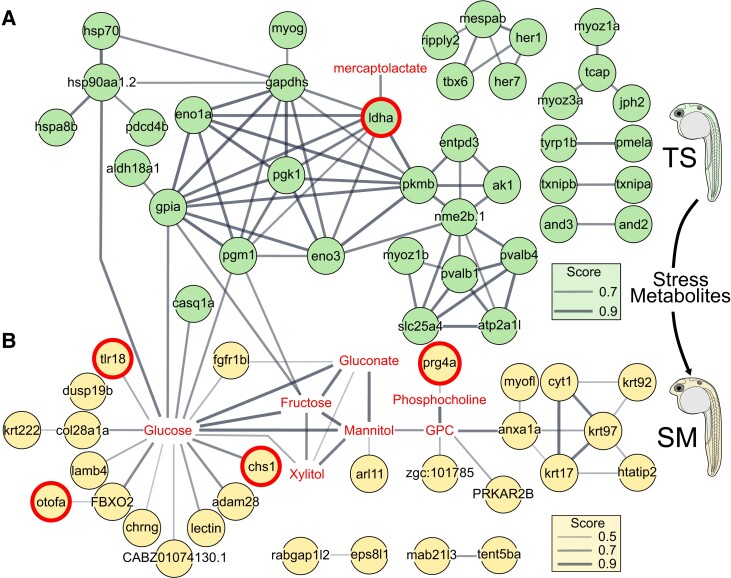
Heat-induced DEGs are functionally connected to stress metabolites, which interact with the transcriptome of receivers. Hypothetical CPI network analysis of significant DEGs (circles) of TS (top row, A) or genes of SM (bottom row, B) and stress metabolite compounds (in red without circle). Line width represents the increasing confidence score with a minimum threshold of 0.7 (TS) or 0.4 (SM). Computed and drawn independently for A) and B) in STITCH using Cytoscape and merged in Inkscape. A) Compound-protein interaction (CPI) enrichment *P* < 0.0001, 64 edges observed vs. 5 expected edges. B) CPI enrichment *P* < 0.0001, 14 CPI edges observed vs. 3 expected. GPC, glycerophosphocholine. Treatments were SM, stress metabolites at 27°C, and TS, fresh medium in thermal stress compared with C, control in fresh medium at 27°C. Genes with red bold circles were used for LAMP measurements. Genes with red bold circles were selected for LAMP measurements based on evidence from multiomic data of their possible involvement in stress propagation and functional links with stress metabolites.

Next, we analyzed the transcriptome of heat stress on zebrafish embryos to identify the molecular routes that lead to the release of SM (Hypotheses 1 and 2, Fig. 1A). Repeated heat stress induced *n* = 369 differentially expressed genes (DEGs) (*P* ≤ 0.01) (Figs. 2A and S3, PCA). Functional enrichments [biological processes (BP), KEGG, and Reactome] were conducted for the top 106 genes (Dataset S1) with strongest support (*P*-adj ≤ 0.05), most of which were up-regulated [*n* = 90 genes with fold-change (FC) > 1.5 and log fold-change (LFC) > 0.58], while a few were down-regulated (*n* = 16 genes). The three genes with largest effect size and LFC > 3 were *atp2a1l*, *crygm2d18*, and *matn3a*. Two paralogs of gamma-glutamylamine cyclotransferase, tandem duplicate (*ggac*t.2 and *ggact.3*), involved in glutathione metabolism, were the two most heat-inhibited genes, with 98% fewer transcripts than in control embryos. Heat stress induced transcriptomic changes in embryonic development (eyes, muscles, and somites), in the heat stress response, and the metabolism of sugars, amino acids, purine, and energy intermediates (ATP and pyruvate, Fig. 2D, Supplementary Results, Fig. S4, and Dataset S1). In addition, network analyses revealed a strong functional interconnectivity between the DEGs of TS [Fig. 3A, compound–protein interaction (CPI) enrichment *P* < 0.0001, 64 edges observed vs. 5 expected edges]. A cluster of co-upregulated genes was assigned to the GO term “metabolism of carbohydrates” (GO:0005975): *pgk1*, *gpia*, *eno1a*, *eno3*, *ldha*, and *gapdhs*. Similarly, another cluster grouped five transcription regulators (GO:0000122) of the GO terms “segmentation” (GO:0035282) and “somite development” (GO:0061053): *mespab*, *her1/7*, *ripply2*, and *tbx6* (Fig. 3A and Dataset S1). Overall, the molecular data at 1 and 4 dpf are in line with a stress response in heat-exposed embryos, which validates our experimental design for studying stress propagation.

### SM induce a localized transcriptomic signature in 1-dpf embryos

We then explored the transcriptome of receivers to infer whether they reacted to SM at the transcriptome level (Hypothesis 3, Fig. 1A). While the transcriptomic response to the SM treatment was less pronounced compared with the whole-body response to heat stress, SM altered 79 transcripts with raw *P* values lower than 0.01 (Fig. 3B and Dataset S1). Respectively, 45 and 15 of these were up- or down-regulated in SM compared with C. The gene *si:ch211-214b16.2* (ENSDARG00000102593, significant after *P* value adjustment) was down-regulated by 99.7% by SM and is likely an ortholog of the gene NOD2 identified as orthogroup 45875at7898 at Actinopterygii level by OrthoDB v10.1 (30). Other than expected, genes belonging to the GO term “chemosensory perception” (GO:0007606) were not significantly enriched (Dataset S1). Instead, the transcriptomic response to SM was related to four wider categories: extracellular signaling, (phospho-) lipid and anion transport, the metabolism of glycosaminoglycan and keratan, but also the immune response involving interleukin cytokines and apoptosis (Fig. 3B, Supplementary Results, Fig. S5A and C, and Dataset S1). The glycosaminoglycan/keratan/keratin terms involved several genes including *krt17*, *krt97*, chitin synthase 1 (*chs1*), carbohydrate (*N*-acetylglucosamine-6-*O*) sulfotransferase 2b (*chst2b*), but also a proteoglycan *prg4a*. SM-responsive genes were also significantly (*P* < 0.0001, 14 CPI edges observed vs. 3 expected) functionally connected in a network (Fig. 3B) which supports the biological relevance of the measured response. Prior work identified the lateral line as a candidate organ activated by chemical cues (31, 32), which led us to compare the expression of genes expressed by SM and in lateral line hair cells. Remarkably, the DEGs in SM are also significantly up-regulated in zebrafish lateral line hair cells in 5-dpf larvae, relative to their expression in neighboring epidermis cells (*z*-score = 8.71505; *P* < 0.0001, Fig. S5D). One interesting gene emerging from this comparison was *xkr8.2* (ENSDARG00000076820), which is the highest up-regulated gene in both hair cells and SM (LFC = 1.68) compared with nonhair cells, and is a scramblase involved in the enriched GO term “membrane phospholipid transport” (GO:0015914, Dataset S1). Overall, our results show that SM deviated the homeostatic molecular response within receivers compared with the control condition, indicative of a stress response and in line with Hypothesis 3 (Fig. 1A).

### Heat stress and SM induce few similar genes and have unique effects in combination

We then compared the transcriptomic profiles in TS and SM to infer the shared genes and functions of the stress responses in donors (Hypothesis 1) and receivers (Hypothesis 3). Nine genes were common to both individual TS and SM conditions compared with control C. Genes up-regulated in both conditions were associated with eye development (*cryba1b*), immune response (*tcima*), chloride transmembrane transport (*best1*), and cell death (*fthl28*). In contrast, *ggact.2*, involved in glutathione metabolism, was inhibited by TS but up-regulated in SM. In the natural environment, if heat stress occurs, SM would increase simultaneously. We expected that heat may alter the response to stress cues (Hypothesis 4). To test this, we therefore explored the transcriptome of the combined TS + SM treatment, which was mainly driven by heat stress as 49% DEGs (78/159) were shared between TS and TS + SM (Figs. 2C and S6A), with functions being largely similar to those in TS (Supplementary Results, Figs. S6B and S7A, and Dataset S1). However, the combined treatment also up-regulated one olfactory gene, the taste receptor olfactory receptor class A related 3 (*ora3*, GO term-wide *P*-adj = 0.0412, Dataset S1). Moreover, 47% of DEGs (75/159 with *P*-adj < 0.05) were uniquely up- or down-regulated in TS + SM condition, suggesting an interaction between the SM and TS factors causing a different response than either factor by itself, with additional functions unique to TS + SM ascribed to developmental processes (eyes, heart, and muscles). Interestingly, this involved apelin signaling (with five genes *acta2*, *si:ch211-286b5.5*, *mef2aa*, *ryr2a*, and *map1lc3cl*; Figs. 2F and S6C and D, and Dataset S1). We additionally compared the treatment TS + SM with SM and TS to determine responses of combined vs. single stressors (Fig. S7B and C and Dataset S1). One particular gene that was significantly altered by TS + SM compared with SM was olfactory receptor C family, b1 (*olfcb1*, LFC = 2.30, *P*-adj < 0.0001), suggesting that heat in combination with SM affects chemosensation. Comparing TS + SM with the TS treatment showed that the addition of SM adds 32.5% novel BP terms, 37.5% Reactome pathways, and 16.7% KEGG pathways (Fig. S7D and E). Overall, our results show that heat stress altered the response to SM, supporting our Hypothesis 4 (Fig. 1A).

### Stress and control media show distinct metabolomic profiles

**Fig. 4.**
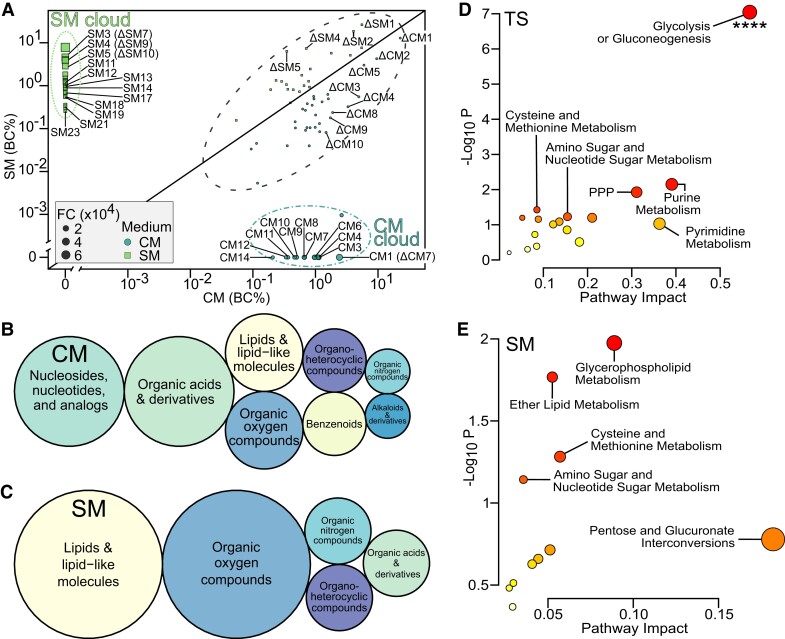
Multiomic data evidence that stress metabolites (SM) differ in classes and functions from control metabolites (CM). A) Correlation plot showing filtered (*n* = 89) masstags that are possible biomarkers of CM (blue circles, bottom right) and SM (green squares, top left) groups. Data are presented as blank-corrected total percent intensity (BC%), which represents the relative concentrations of the compounds in the medium sample. Masstag inside the black dotted ellipse are unlikely to be biomarkers of SM or CM since they are present in both conditions. Masstags inside the dot-dashed blue and dashed green ellipses represent CM- and SM-specific biomarkers, respectively. FC, fold-change between SM and CM where the numerator is the medium with the highest concentration for each compound. Labels show the masstags with possible hits among the top 23 compounds of SM, top 14 compounds of CM based on fold-change (see Table S3 for associated chemicals), and top 10 compounds based on difference between SM and CM (delta Δ identifiers). Superclasses of representative hits for the biomarkers of B) CM and C) SM. Bubble sizes are proportional to counts of superclasses per medium type. Candidate cause-and-effect pathways derived from multiomic analysis of KEGG pathways integrating the representative hits of the SM with the DEGs of D) TS or E) SM. The top five most evident pathways are given within plots, respectively. Significant terms are shown by **** (*P*-adj ≤ 0.0001). PPP, pentose phosphate pathway. See Dataset S2 for class-sorted compounds.

Next, we asked whether heat-stressed individuals release SM within their environment that differ from that of undisturbed animals and could be responsible for stress propagation (Hypothesis 2, Fig. 1A). For this purpose, we explored the metabolome released in the media by heat-stressed vs. control embryos and the relative concentrations of the metabolites they contain (Fig. 4A). The metabolomic profile of the SM contained 89 metabolites which were matched to 658 possible compounds (Figs. 4A and S8 and Dataset S2). Figure 4A represents the blank-corrected concentrations of compounds in SM and CM, which showed that metabolites were either present in both media in varying concentrations or were uniquely secreted in either CM or SM (dubbed “CM cloud” and “SM cloud,” Fig. 4A and Table S3). Two different masstags were assigned to 3′-mercaptolactate (SM12 and SM21). Several a priori–defined candidate chemicals for SM (i.e. from alarm/disturbance cue studies, Table S2) were unexpectedly more concentrated in control medium compared with stress medium, such as hypoxanthine-3-*N*-oxide (C_5_H_4_N_4_O_2_, for which we found hypoxanthine C_5_H_4_N_4_O), trigonelline or homarine, cysteine, or acetylcholine (Table S3 and Dataset S2). Representative compounds of each identified masstag were used for functional subclass and KEGG pathway enrichments (Fig. S9 and Dataset S2), which revealed that CM were mainly associated with amino acids (e.g. CM8 proline and CM14 l-tyrosine), catechols, purine ribonucleoside monophosphate (e.g. CM6 adenosine monophosphate), and dipeptides. SM in contrast were mostly associated with sulfur-containing carbothioic S-acids (organosulfur compounds) and lipids including *O*-acylglycerol-phosphates, fatty acids, and glycerophospholipids (Fig. S9). The representative SM compounds were mainly ascribed to lipid molecules and “organic oxygen compounds”, whereas control medium contained molecules which had a more diverse chemical classification and were mainly “organic acids and derivatives” and “nucleosides, nucleotides, and analogs” (Fig. 4B and C, Table S3, and Dataset S2). Several compounds did not have a ChemOnt classification but were likely proteins (SM1, SM4, SM19, and SM23) or polyamines (SM7). Overall, the metabolomic data showed that CM (i.e. metabolites within the medium collected from C) differ from SM (i.e. metabolites within the medium collected from TS).

### Multiomic analysis uncovers sender–receiver gene–metabolite candidate cause-and-effect pathways

We then aimed to investigate the molecular chain of events between senders and receivers of SM by searching for functional links between SM and the transcriptomes of donors and receivers. Since (i) the DEGs of heat-stressed donors (TS vs. C) putatively lead to the synthesis and/or release of SM, which (ii) may in turn have regulated the transcriptomic signature in receivers (DEGs from SM vs. C), we explored this possibility using joint pathway and STITCH analyses (Dataset S2). The joint pathway analysis evidenced that donors significantly enriched the “glycolysis/gluconeogenesis” pathway and compounds (Fig. 4D). Receivers, in contrast, enriched, albeit with weak evidence (raw *P* ≤ 0.07 but FDR > 0.05), glycerophospholipid, ether lipid, cysteine and methionine, and amino sugar metabolism pathways (Fig. 4E and Dataset S2). The hypothetical CPI network evidenced a strong connectivity of SM with DEGs of either TS (64 CPI edges observed vs. 5 expected edges, *P* < 0.0001) or SM (14 CPI edges observed vs. 3 expected) (Fig. 3). For instance, lactate dehydrogenase A4 (*ldha*) was linked to 3′-mercaptolactate in donors (Fig. 3A), whereas in receivers, many genes, including toll-like receptor 18 (*tlr18*), chitin synthase 1 (*chs1*), and otoferlin A (*otofa*), were linked to glucose, while proteoglycan 4a (*prg4a*) was associated with phosphocholine (Fig. 3B). Altogether, our results show that there may be functional links between the molecular response of directly heat-stressed metabolite donors and receivers.

### Both heat stress and heat-induced SM alter development and stimulus-induced behavior

**Fig. 5.**
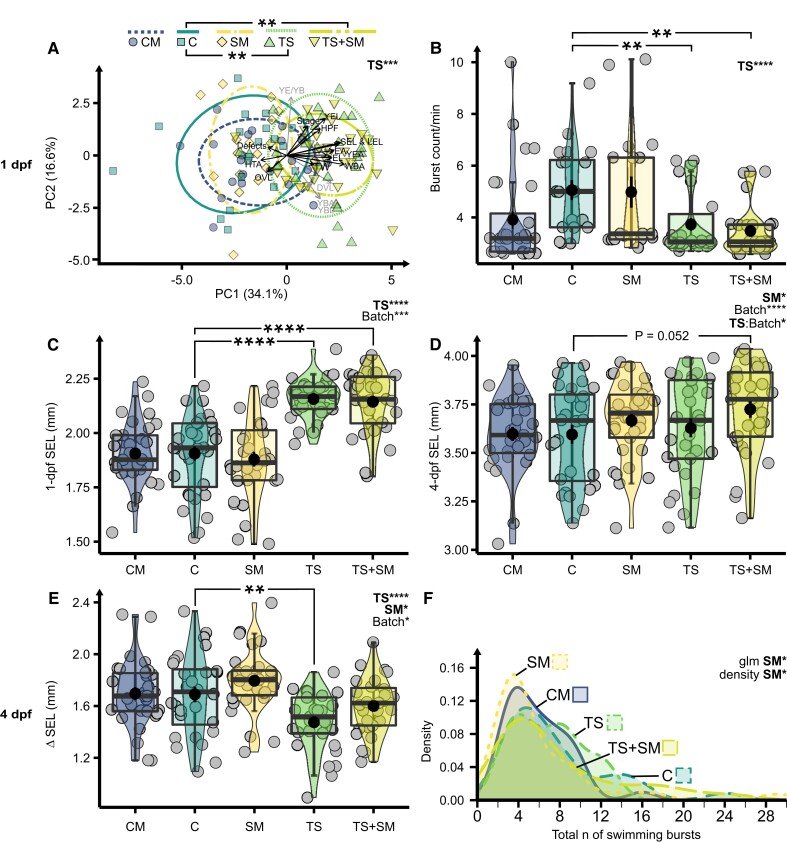
TS and SM alter the development and behavior of zebrafish embryos. A) Principal component analysis (PCA) of 1-dpf morphology. HPF, final stage in hours post fertilization; Stage, % in segmentation or pharyngula; EA, eye area; EL, eye length; DVL, dorsal–ventral length; HTA, head–trunk angle; LEL, longest embryo length; OVL, otic vesicle length; SEL, shortest embryo length; WBA, whole-body area; YBA/L, yolk ball area/length; YEA/L, yolk extension area/length; YE/YB, yolk extension/yolk ball ratio. Individual variables significantly altered by TS are shown by black arrows. B) Burst count per minute in active 1-dpf embryos. C) SEL (mm) increment from 1 to 4 dpf. D) Mean acceleration (m/s^2^) of the first complete burst following the first and third touch stimuli. E) Density distribution of total burst count and total distance in cm F) after three touch stimuli. Effects of TS and SM across the two-way factorial design (C, SM, TS, and TS + SM) were computed using PERMANOVA A), ANOVA B–D, F), or negative binomial generalized linear model (E, confirmed by a density test) with significant predictors and covariates (“batch”) reported on top-right corners. Pairwise tests with FDR *P* value correction compared CM to C and SM, or C to SM, TS, and TS + SM, with significant comparisons shown by horizontal bars. *P ≤ 0.05, **P ≤ 0.01, ***P ≤ 0.001, and ****P ≤ 0.0001. Treatments were CM, control metabolites at 27°C; C, control in fresh medium at 27°C; SM, stress metabolites at 27°C; TS, fresh medium in thermal stress; and TS + SM, stress metabolites in thermal stress.

After having unraveled the molecular mechanisms underlying stress propagation, we asked what were their consequences on the overall biology of animals by monitoring fitness-relevant phenotypic traits (development and behavior) over the course of development in directly heat-stressed embryos and SM receivers. We expected that heat and SM were stressors that could cause changes in escape and startle behavioral responses, which are standard measurements to indicate anxiety and changes in sensory and motor functions (33–35). Heat stress (*F* = 30.1, *R*^2^ = 0.25, *P* = 0.001), but neither SM (*F* = 0.16, *P* = 0.7610) nor the interaction term (*F* = 2.34, *P* = 0.1320), altered the phenotype of 1-dpf embryos (Fig. 5A and Table S4). Post hoc tests confirmed that thermal stress significantly altered embryo growth, reaching the pharyngula stage earlier (*z* = −2.67, *P* = 0.0076, Figs. 5C and S10A and B). Conversely, SM had no effect on growth at 1 dpf. However, an ANOVA of the two-way model terms showed that 4-dpf zebrafish embryos grew significantly longer in the presence of SM (*t* = 4.80, *P* = 0.0304, small effect size = 0.35) but not under thermal stress (*t* = 1.91, *P* = 0.1692) nor the interaction term (*t* = 0.11, *P* = 0.7353, Fig. 5D). An ANOVA revealed that both heat and SM significantly altered the increment in growth (ΔSEL) between 1 and 4 dpf, but in opposite directions (Fig. 5E and Tables S5 and S6). Heat-exposed embryos had a lower ΔSEL (*t* = 20.99, *P* < 0.0001, large effect size = 0.81), whereas those experiencing SM accelerated growth from 1 to 4 dpf (*t* = 6.65, *P* = 0.0112, small effect size = 0.43) and slightly surpassed the TS treatment in average final length at 4 dpf (Fig. 5D and E). Heat stress (*t* = 18.15, *P* < 0.0001), but neither SM (*t* = 0.29, *P* = 0.5891) nor their interaction (*t* = 0.07, *P* = 0.7872), significantly reduced the startle response of 1-dpf zebrafish embryos (Fig. 5B and Tables S7 and S8). Heat also slowed mean acceleration (*t* = 4.26, *P* = 0.0412, small effect size = 0.34), and TS marginally reduced mean speed compared with C (*t* = −2.74, *P* = 0.0212) in the touch-evoked swimming response of 4-dpf larvae (Fig. S11B and C). Larvae incubated in SM, however, swam fewer burst counts compared with fresh medium (*t* = −2.1, *P* = 0.0381, Fig. 5F). Since the metabolomic analysis supported our hypothesis that SM from stressed animals are different compared with CM from undisturbed controls, we explored the consequences of SM vs. CM at the phenotypic level. We found that SM tended to induce less hatching (Fig. S10D) and tended to have altered startle responses at 1 dpf (Figs. 5B and S10F and Table S8) and swimming patterns at 4 dpf (Fig. S11D) compared with CM. Overall, alterations in fitness-relevant phenotypes confirmed that both TS and SM induced stress responses in zebrafish embryos and that CM and SM have different phenotypic consequences. See Supplementary Results for an extensive description of phenotypic data.

### SM also exist in group-raised nonlaboratory embryos

Laboratory strains of zebrafish may perform differently to their wild counterparts, raising questions about the ecological relevance of findings using less-plastic laboratory-adapted strains (22, 36, 37), and embryos in clutches may react differently to social cues to single embryos in tubes due to their social nature (38, 39). In line with this, in our laboratory AB line experiments, we found a localized response to SM and a lower phenotypic response than we found previously (16) using pet store (PET) line embryos (Fig. S12). Therefore, we aimed to explore the effects of SM in social groups of a more genetically diverse strain, that is, in settings that better match the natural conditions of zebrafish to gain further insights about the ecological relevance of our findings. For this purpose, the expression of a subset of genes (*chs1*, *ldha*, *ora3*, *otofa*, *prg4a*, and *tlr18*) was measured in an independent confirmatory experiment utilizing nonlaboratory (PET line), group-raised zebrafish embryos. These genes were selected based on the evidence of their involvement in stress propagation gathered from RNA-seq data and functional links with possible SM (see previous sections). We expected to find similar molecular responses to SM in group-raised outcrossed zebrafish embryos, perhaps with different magnitudes of gene expression due to changes in density ratios and concentrations of SM.

First, *ldha*, overexpressed in both TS and TS + SM in the RNA-seq data and functionally linked to one possible SM (3-mercaptolactate), was not altered in response to SM (*t* = −0.14, *P* = 0.8960, Fig. 2H and Table S9). Two candidate genes involved in sensory perceptions (*otofa*, which we identified as a gene of interest expressed in SM and also expressed in lateral line hair cells of 5-dpf larvae, see Dataset S1) and immunity (*tlr18*, which we selected because SM altered immunity-related functions) were (nonsignificantly) up-regulated by SM (Fig. 2J and L and Table S9). SM significantly up-regulated *prg4a* (which was selected for its interaction with phosphocholine SM in receivers; *t* = −8.01, *P* = 0.0026, |*d*| = 6.52, “huge” effect size), with all of these genes’ expression patterns matching the RNA-seq data of the AB line zebrafish. In contrast, *chs1* was identified as an important keratan sulfate-related gene and was significantly down-regulated (*t* = 3.08, *P* = 0.0487, |*d*| = 2.18, “huge” effect size, Fig. 2G and K and Table S9) in this experiment, contrasting with significant up-regulation in SM of the AB line experiment (LFC = 1.64). One chemosensory candidate gene, *ora3*, could not be detected in the SM treatment, suggesting receptor depletion in embryos exposed to SM (Fig. 2I).

## Discussion

While the concept of stress propagation between disturbed animals is well established in certain contexts, such as predation (15), we lack knowledge about the mechanisms and consequences of social stress transmission (18) and about the extent of this phenomenon upon abiotic stress and in early life stages. Therefore, we aimed to elucidate the molecular aspects of heat-induced stress propagation in zebrafish embryos. Our contribution demonstrates previously unidentified cues responsible for stress propagation along with the transcriptome and phenotypes of abiotically stressed senders and receivers.

We first confirmed that repeated heat peaks alter a range of different aspects of the biology of zebrafish embryos. In our study, heat stress altered molecular profiles leading to behavioral and developmental changes and earlier hatching. Contrasting with studies reporting hyperactivity upon heat stress (40, 41), zebrafish embryos were hypoactive, which likely reflects differences in the temperature regime we used. The up-regulation of heat shock protein (HSPs) transcripts and cortisol confirmed that repeated heat stress induced a (cellular and heat) stress response independent of faster development (42, 43). However, the induction of HSP70 found at 1 dpf (16) was no longer present in 4-dpf larvae having experienced 4 days of repeated heat peaks, which suggests either attenuation of the heat shock response (9, 10) or habituation to higher temperatures. One limitation of our study is that we only measured protein levels of HSP70, but a more comprehensive picture could be obtained by analyzing other heat-responsive chaperone proteins. Heat stress induced glucose, pyruvate, and ADP/ATP metabolism, indicating mobilization of energy reserves. Heat stress altered developmental transcriptome dynamics. As a consequence, heat-stressed embryos were older and grew longer at 1 dpf, in accordance with previous studies (4, 16, 44). However, we observed a subsequent slowdown of development from 1 to 4 dpf conforming to the temperature–size rule stating that fish growing faster during early stages become smaller adults (45). Of note, future studies should use additional stage-matched controls to decipher the involvement of heat-induced development vs. heat-induced behavioral changes—as of now, we do not know yet whether the effects of TS are a consequence of heat stress or heat-accelerated development. Overall, using common broad definitions of stress as deviations to homeostasis (26–29), our data showed that heat induced stress in embryos compared with controls, allowing us to investigate stress propagation toward conspecifics.

Only a few prior studies have characterized stress-induced whole-body metabolome profiles in fish (46–48), but none yet have focused on cue secretion into the environment. Here, we used metabolomics to explore the hypothesis that stressed animals release stress-induced metabolites in their environment (Hypothesis 2, Fig. 1A). A major finding in our study was that heat induces the release of SM into the medium that are distinct from CM released by unstressed embryos. The multiomic compound–protein interactome evidenced functional links between transcriptomes and the metabolome containing SM released into the medium. Interestingly, several candidate chemicals (e.g. trigonelline, homarine, and hypoxanthine-3-*N*-oxide) previously identified as alarm cues (49, 50) were more concentrated in the control medium. In contrast, SM were mainly ascribed to two compound superclasses: “organic oxygen compounds” and “lipids and lipid-like molecules.”

Carbohydrates within SM may originate from heat-induced metabolic activity such as glycolysis (51). The multiomic analysis highlighted that the pentose phosphate pathway (PPP, including deoxyribose, *gpia*, and *pgm1*), a major cellular redox mechanism (52), was altered in response to heat. Organosulfur compounds (such as 3-mercaptolactate or 2-oxo-4-methylthiobutanoic acid) were a main component of the organic oxygen compound classification. Multiomic analyses of SM with receiver DEGs revealed “cysteine and methionine metabolism” as the third most important altered pathway, lending support to a functional relationship between secreted organosulfur compounds and receiver responses. 3-mercaptolactate is part of the cysteine transamination pathway and synthesized by the heat stress-induced DEG lactate dehydrogenase A *ldha* (53). Lactate dehydrogenase was likewise found to be up-regulated by heat stress in rockfish *Sebastes schlegelii* (46). Organosulfur compounds may function as cues in chemical ecology, for instance, in fox urine (54) and cat urinary pheromones (55), and therefore warrant further study.

In addition, SM predominantly contained a range of lipids, including fatty acyls, glycerolipids, sphingolipids, glycerophospholipids, and steroid esters. The multiomic analyses found “glycerophospholipid metabolism” and “ether lipid metabolism” as the two major KEGG pathways enriched between SM and receiver transcriptomes. Previous lipidomic studies demonstrated a role of lipids, particularly sphingolipids, in protecting the cell from heat damage and acting as messengers (56, 57). Differential vulnerability of lipid classes to reactive oxygen species leads to temperature-controlled membrane lipid remodeling (58, 59). Two notable glycerophospholipids among SM were phosphocholine and glycerophosphocholine, which is known from tissues of heat-stressed olive flounder *Paralichthys olivaceus* (60) and may originate from temperature-induced breakdown of phosphatidylcholine in the cell membranes (51). A possible role for lipids in chemical communication is likewise documented (61). For instance, mating in reptiles may depend on epidermal skin lipids (62), and migratory behaviors in sea lamprey are regulated by larval-released fatty acid derivatives (63). Lastly, in zebrafish embryos, these lipids may also originate from the yolk sac, being the most common membrane lipids (64). Since membrane lipids such as phosphatidylcholine are known not only as cellular signaling molecules, but also as conveying group identity between newly hatched catfish (*Plotosus lineatus*; 65), this supports a role for heat-mediated change in composition of membrane lipids as heat stress-related signaling molecules. For instance, *xkr8.2*, active within zebrafish lateral line hair cells (66)—which plays a role in chemical communication (32)—is overexpressed in response to SM. Its human ortholog *xkr8* flips phospholipids between the inner and outer membrane layers (67), so it could be used by receivers to interact with lipid SM. Further compounds functioning as SM could be peptides or proteins (68), which we could not characterize further. It is worth noting that our metabolomics analysis is exploratory, and we acknowledge that without the use of internal standards, we have not definitively identified individual metabolites. Still, our metabolomic data enabled us to provide evidence for chemical classes of SM—in addition to possible candidate molecules—released by stressed embryos into their environment. Further mass spectrometry studies are needed to definitively identify the compounds, to discover more chemicals that act as SM, and also to discriminate the involvement of heat-induced developmental effect vs. heat-induced metabolic shifts in the synthesis of candidate SM. Future studies should test the direct effects of SM we identified to validate their involvement in stress propagation.

SM released by heat-stressed donors induced stress in naive receiver embryos, indicative of a positive feedback loop (Fig. 1A). Molecular and phenotypic effects included accelerated development and impaired behavioral activity, as previously found for SM (16) and alarm cues (24). These phenotypic effects were similar to those induced by heat itself, but developmental acceleration caused by SM had a later onset at 4 dpf. SM also initiated a suite of cellular signaling pathways different from those induced by heat. The most significantly down-regulated gene in SM receivers was *si:ch211-214b16.2* (NOD2 ortholog) which is involved in intracellular signal transduction (69) and the activation of apoptotic and immune pathways (70, 71). Further immune and apoptosis responses were induced via *fthl28* and *tlr18*. *tlr18* is a fish-specific toll-like receptor expressed in the skin, regulated by infection challenge and lipopolysaccharides (72), and may be homologous to the mammalian *tlr1* which binds lipopeptides (73). Therefore, *tlr18* may be directly responsive to lipid SM present in the medium, which we could independently confirm in group-exposed PET line zebrafish. Corroborating these results, brain transcriptomes of three-spined stickleback *Gasterosteus aculeatus* exposed to predator and alarm odors were also enriched for apoptosis, immune, and signaling pathways (74). Moreover, several genes related to development such as keratins were activated by SM, which may explain the observed developmental acceleration in this treatment. In addition to hypoxanthine 3-*N*-oxide (C_5_H_4_N_4_O_2_ for which we here found C_5_H_4_N_4_O to be more prevalent in the control condition), *Schreckstoff* is also known to contain other components such as extracellular polysaccharides (glycosaminoglycans), presumably but not exclusively chondroitin sulfate, and mucin proteins (75). The second major glycosaminoglycan in zebrafish is keratan sulfate (76), which is bound to proteoglycan, another component of the mucus layer next to mucins. In this contribution, we found that phosphocholine and sugars secreted from heat-stressed embryos significantly up-regulated proteoglycan 4 (*prg4a*) and altered two transcripts involved in keratan sulfate biogenesis (*chs1* and *chst2b*) in receiver embryos, both in AB and PET line experiments. This lends support to proteoglycan 4 and keratan sulfate being signaling components alike to *Schreckstoff*, and since this was observed in receivers, but not senders, of SM, it could mean that naive receivers may themselves propagate the release of *Schreckstoff*-like social anxiety cues to further embryos in a developing clutch. Of note, the absence of expression of the chemosensory receptor *ora3* in embryos group-exposed to SM suggests receptor depletion upon involvement in SM detection. While comparing genes expressed in SM vs. sensory hair cell genes also pinpoints the possible involvement of olfactory-mediated pathways in response to SM, more work is needed to confirm its role by using stages at which the olfactory system is fully developed. It is also important to note that studies on chemical cues should utilize chemicals from undisturbed controls (15). We used such control with the additional CM condition, which showed that the chemicals released by unstressed (CM) vs. stressed (SM) individuals induce different phenotypic outcomes, in line with our previous study (16). While future studies should also compare SM vs. CM through transcriptomics to address this limitation in our current work, we anticipate that there would also be differences in the gene expression patterns as supported by consistent differences in key antioxidant and immune genes in our prior work (16).

Outside of the laboratory, high-density population scenarios such as developing fish clutches exposed to heat would experience SM simultaneously with heat, leading to combined exposure. Here, the combined treatment of heat and SM from heat-exposed embryos revealed that these larvae grew the most and also had the lowest behavioral activity. While heat was the predominant driver of this transcriptome, it also showed unique responses compared with the heat-only treatment, catalyzed through the addition of SM. These involved differential expression of two chemosensory (vomeronasal receptor-like) genes, *ora3* and *olfcb1*, and genes involved in developmental acceleration via five genes in the apelin signaling pathway. Apelin signaling is a type of environmental signal processing with a wide array of physiological effects such as angiogenesis, renal fluid homeostasis, energy metabolism, immune response, embryonic development, and the neuroendocrine stress response (77), which hints at activation of the sympathetic nervous system (SNS) (78) and the emergence of chronic stress (79).

**Fig. 6.**
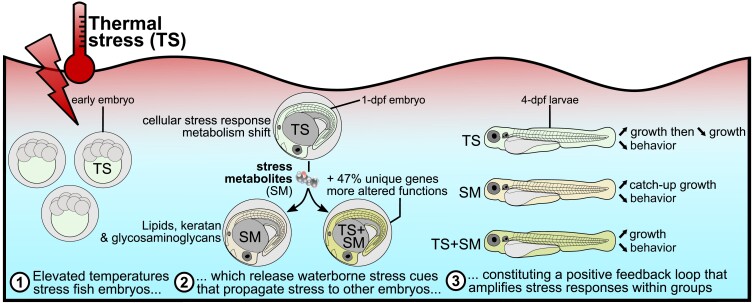
Conceptual summary of stress-induced communication mediated through SM in zebrafish embryos.

The basic principle of stress communication dates back to the 1930s with von Frisch's discovery that alarm cues from injured fish scared conspecifics (80, 81), which was then also shown in noninjured animals (82). However, despite decades of research, most studies on chemical cues heavily focused on behavioral and physiological endpoints monitored in animals disturbed by biotic stressors, mostly related to predation (15, 83). With the exception of one study by Hazlett in 1985 in crayfish exposed to heat (19), and our pilot studies using pH in marine species (17) and heat stress in zebrafish (16), there is little evidence about (i) whether climate change–related abiotic stress can induce stress propagation, (ii) their prevalence in embryonic and larval stages, and (iii) the underlying molecular mechanisms of stress propagation. We here address these gaps by investigating the molecular mechanisms and fitness-relevant consequences of stress propagation in heat-stressed fish embryos. We showed that heat effects have been propagated to receivers through SM, inducing similar phenotypic outcomes and molecular mechanisms related to growth acceleration, but also some characteristic differences to the direct stress response such as the absence of cortisol and the use of different signaling pathways (Fig. 6). Our findings support the hypothesis that abiotically stressed animals can chemically propagate stress responses in conspecifics (Fig. 1A), leading to fitness-relevant consequences on development and behavior (Fig. 6). We therefore anticipate that such positive stress feedback loops warrant further study due to the ever-increasing occurrences of diel and seasonal heat events. Importantly, we evidenced the prevalence of stress propagation in a social context using a more natural zebrafish strain which supports the relevance of our findings as a framework basis for future ecological studies on chemical stress propagation in aquatic animals.

## Materials and methods

### Experimental design

Zebrafish embryos were collected by breeding adult zebrafish at the University of Hull. Embryos were collected in the morning, cleaned in fresh 1X E3 medium, and rinsed by bleaching. Embryo stages were measured in hours post fertilisation (hpf) according to Kimmel et al. (84). Starting from the blastula period (∼3.3 hpf), two temperature protocols were used, either (i) a control constant temperature of 27°C or (ii) repeated temperature fluctuations from 27 to 32°C. While departing from realistic heatwaves, this temperature protocol contained maximal repeats of sublethal +5°C heat peaks during crucial times of embryogenesis and larval development. Of note, our results have to be interpreted with respect to constant darkness delaying the normal zebrafish embryonic development (85). A factorial design for the two factors “temperature” and “SM” (Fig. 1B) yielded four experimental treatments: fresh medium at 27°C (control C), fresh medium with thermal stress (TS), medium containing SM at 27°C (SM), and medium containing SM with thermal stress (TS + SM). A fifth condition CM had control metabolites contained within the medium collected from C. We measured five different endpoints at different time points, which yielded (i) cortisol, (ii) transcriptomic, (iii) metabolomics, (iv) multiomic, and (v) phenotypic data (Fig. 1C). We combined transcriptomic and metabolomic data, to explore the mechanistic basis of stress propagation, with fitness-relevant phenotypic responses, to understand its consequences at the whole-body level. The different time points were selected to account for the specific onset of each measured response during embryogenesis. For endpoints i–v, embryos were exposed in isolation in individual PCR tubes. Additionally, we aimed to evaluate the validity of our findings by testing their repeatability in different experimental settings by using a different zebrafish strain and altering the temperature stress and fish density protocols. For this purpose, (vi) the effects of SM on gene expression were measured in nonlaboratory PET line embryos exposed by groups of 20 embryos in petri dishes (Fig. 1D). For molecular endpoints, embryos were pooled for data collection, while phenotypic data are reported for each individual embryo. Briefly, medium was prepared according to published standards (86), and putative metabolites were obtained by pooling medium conditioned by heat-stressed (stress medium containing SM) or control (control medium containing CM) embryos on the day prior. Studies on stress cues should use odors from undisturbed individuals as control (15). Therefore, we included CM released by control embryos in our experimental design for phenotypic data. However, we opted to perform the RNA-seq using fresh medium (condition C) as the appropriate control for the two-way factorial design including SM, TS, and TS + SM treatments in order to remove confounding effects of signaling molecules and their induced pathways in receivers from CM. Refer to Supplementary Methods for further details on fish handling methods and medium and temperature protocols.

### Endpoint 1: cortisol and HSP70 response to heat and heat-induced metabolites in 4-dpf embryos

We first aimed to confirm that our heat stress protocol induced a stress response in zebrafish donors, as a condition to study stress propagation. Cortisol is a reliable stress biomarker (43) that we used, along with the heat-inducible HSP70 (87), to infer whether heat-exposed embryos were stressed. HSP70 was used as a stress biomarker based on its well-established induction in fish through heat (87), including by environmental variation in temperature (88–90). We previously quantified HSP70 in 1-dpf embryos (16). Since cortisol is not stress responsive before 2–4 dpf (91, 92) and our heat stress protocol does not induce a cortisol spike at 1 dpf (16), we aimed to determine the optimal time point to assess the cortisol stress response; we followed the dynamics of cortisol levels in control embryos at 1, 2, 3, and 4 dpf. For this purpose, control embryos were incubated in E3 medium in clutches at 27°C with natural (∼12:12 dark:light h) light conditions. Embryos were pooled into groups of 40 embryos per sample (*n* = 3 pooled samples per time point). After optimization for ELISA assay, zebrafish embryos were exposed in individual wells to C, CM, TS, and SM treatments from 0 to 4 dpf, until 60 embryos per biological replicate were obtained for each treatment (*n* = 3 samples per treatment, total of 180 embryos per treatment). Cortisol and HSP70 protein levels were then quantified in 4-dpf larvae.

### Endpoint 2: transcriptomic response to heat and heat-induced metabolites in 1-dpf embryos

To explore the molecular basis of stress propagation, we needed to identify the genes involved in the release of SM in heat-stressed donors and their detection in receivers. Since our pilot study confirmed that SM induced gene expression changes at 1 dpf (16), we used RNA-seq in 1-dpf zebrafish embryos. The transcriptomic response to thermal stress and SM was characterized by individually exposing zebrafish embryos to treatments C, TS, SM, and TS + SM for 24 h. At the end of the exposure, viable zebrafish embryos still in their chorion were pooled into groups of 20 and processed for RNA-seq (*n* = 3 biological replicates of 20 embryos, i.e. total of 60 embryos per treatment). Embryos were pooled for sequencing to increase statistical power by reducing noise from individual variation in gene expression (93). Reads were quality processed, annotated, and analyzed for DEGs, either as a complete data set or by subset of candidate GO terms, and for functional enrichment of BP, KEGG, and Reactome pathways. Since the zebrafish lateral line system is a candidate organ for chemical stimulus perception, we additionally compared the relative expression between hair cells and adjacent nonhair cells, of the SM DEGs vs. other genes expressed in zebrafish lateral line system hair cells from the data set of Elkon et al. (66).

### Endpoint 3: exploratory metabolomics characterization of heat-induced SM

We performed an exploratory characterization of the putative metabolites released by embryos into the medium upon thermal stress. We characterized metabolites released upon thermal stress. Zebrafish embryos were exposed for 24 h. The protocol and medium samples resulted from our previous study (16) where the heating protocol consisted of 13 heat peaks followed by a recovery period of 7 h and 45 min at 27°C until 24 h of treatment were reached and medium would therefore not contain short-lived volatile cues. Media from donor embryos raised in C (i.e. control medium containing CM) or TS (i.e. stress medium containing SM) were collected for metabolite identification. To account for contaminants in the E3 solution and microbial compounds, a blank was prepared by incubating fresh medium without embryos for 24 h at 27°C. LC–MS/MS analysis was performed at the Metabolomics & Proteomics Facility of the University of York. After data preprocessing and blank correction, masstags filtered for signal-to-noise threshold were retained as possible biomarkers of CM or SM (Fig. S1A). These blank-corrected relative concentrations of compounds were used to compare the profiles of stress vs. control media. Possible biomarkers were compared using their unique *m*/*z* masses (± 0.0005 *m*/*z*) to known metabolites, including candidates (Table S2), from publicly available metabolomic databases. Possible biomarkers of either CM and SM groups were then functionally enriched for KEGG pathways and chemical subclasses.

### Endpoint 4: multiomic analysis

To better connect the genetic responses to heat stress by donors and secreted metabolites with the response to these SM by receivers, transcriptomic and metabolomic data were integrated into joint analyses using joint pathway analyses and hypothetical CPI. We reasoned that DEGs in TS may lead to the synthesis and release of SM which would initiate a transcriptome response in receiver embryos. Hence, both the joint pathway and hypothetical CPI analyses were performed by combining the SM with DEGs of either SM or TS.

### Endpoint 5: phenotypic response to heat and heat-induced metabolites in 1-dpf and 4-dpf embryos

A range of phenotypic parameters was measured in zebrafish embryos at 1 dpf and 4 dpf in all five treatments, both within and outside of their chorions after dechorionation. All conditions were tested twice on two independent batches at different dates, using each time one embryo clutch for all treatments to limit batch effects. At 1 dpf, embryos were imaged (Fig. S1B), and light-induced startle responses were recorded. At 4 dpf, larvae were imaged, and touch-evoked behaviors were videoed. Images and videos were randomized for blind analysis. Escape and startle behavioral responses are standard measurements that can indicate anxiety and changes in sensory and motor functions (33–35) and were used to evaluate the effects of treatments on fitness-relevant behavioral outcomes.

### Endpoint 6: loop-mediated isothermal amplification characterization of candidate genes in response to heat SM in outcrossing group-exposed embryos

Finally, we aimed to confirm the relevance of our findings by evaluating the mechanisms of stress propagation in different settings that better match the natural conditions of zebrafish, by utilizing a group exposure of a more genetically diverse strain. For this purpose, outcrossing PET line zebrafish were exposed in groups (3 sets of 20 embryos each) in petri dishes for 24 h to either fresh E3 medium or SM (*n* = 60 embryos per sample, 250 μL medium per embryo). SM were obtained from donors experiencing constant thermal stress of 32°C from 0 to 1 dpf. Total RNA was extracted from four such pools per treatment as previously described to quantify *chs1*, *ldha*, *ora3*, *otofa*, *prg4a*, and *tlr18* using fluorometric loop-mediated isothermal amplification (LAMP) technology, which we chose for its cost-effective and rapid, accurate quantification of gene expression (94). These genes were selected based on evidence from multiomic data of their possible involvement in stress propagation and functional links with SM.

## Supplementary Material

pgad137_Supplementary_Data

## Data Availability

The raw molecular and phenotypic data are available on Zenodo [DOI: 10.5281/zenodo.7308630 (95)], along with the custom code to process and analyze the data. The RNA-seq data is available under the GEO accession number GSE220546 (BioProject PRJNA910181).
