# Supplementary material for: Heat induces multiomic and phenotypic stress propagation in zebrafish embryos
Source: PNAS Nexus. 2023 May 23;2(5):pgad137. doi: 10.1093/pnasnexus/pgad137 (PMC10205475; doi:10.1093/pnasnexus/pgad137)
Supplement: pgad137_Supplementary_Data [file pgad137_supplementary_data.zip › PNASNEXUS-PNASNEXUS-2022-01130-s01.pdf]

PNAS nexus

Supporting Information for

Heat induces multi-omic and phenotypic stress propagation in zebrafish embryos.

Lauric Feugere[1], Adam Bates[1,2], Timothy Emagbetere[1], Emma Chapman[1], Linsey E. Malcolm[3], Kathleen Bulmer[3], Jörg Hardege[1], Pedro Beltran-Alvarez[3] & Katharina C. Wollenberg Valero[1,4]*

[1]Department of Biological and Marine Sciences, University of Hull, Cottingham Road, Kingston upon Hull, HU6 7RX, United Kingdom.
[2]current address: Wellcome Sanger Institute, Wellcome Genome Campus, Hinxton CB10 1SA, United Kingdom
[3]Biomedical Institute for Multimorbidities, Centre for Biomedicine, Hull York Medical School, University of Hull, Cottingham Road, Kingston upon Hull, HU6 7RX, United Kingdom.
[4]School of Biology and Environmental Science, University College Dublin, Belfield, Dublin 4, Ireland.

*Corresponding author: Katharina C. Wollenberg Valero.
Email: katharina.wollenbergvalero@ucd.ie

This PDF file includes:

Supporting text: Supplementary Methods and Supplementary Results
Figures S1 to S12
Tables S1 to S12
SI References

Other supporting materials for this manuscript include the following:

- One submission to Zenodo (DOI: 10.5281/zenodo.7308630)
    - Dataset S1: results from RNA-seq analysis
    - Dataset S2: results from metabolomic analysis
    - Data analysis files and code
        - Code for RNA-seq read file processing
        - R code for data analysis
        - Raw datasets of phenotype data (morphology, behaviour, hatching, survival)
        - Raw datasets for molecular data (cortisol, HSP70, LAMP, gene counts, *DESeq2* results)

- The RNA-seq data published with the accession number GSE220546

Supplementary Methods

Fish husbandry, breeding, and zebrafish embryo handling methods.

Zebrafish embryos from endpoint 3 (metabolomics) and 6 (LAMP) were obtained from a stock of breeders raised in-house for several generations after purchase from a local pet store supplier (PET). Zebrafish embryos used in endpoints 1 (cortisol), 2 (RNA-seq), and 5 (phenotype) were obtained from a breeding stock of adult zebrafish (AB strain) from the University of Sheffield and the University of Cambridge. The reason for this was to limit genetic variation in the embryos sampled for RNA-sequencing by using highly genetically stable embryos from the AB zebrafish line. Fish breeders were maintained at the University of Hull in a temperature-controlled room kept at 27°C with 14:10 light:dark cycle. Fish were fed twice a day an alternate diet of mini-bloodworm, daphnia, and dried flakes. Fish were maintained in appropriate male-to-female ratios for breeding purposes and the fish population was renewed by inhouse breeding.

Breeding was completed by placing plastic trays half-filled with marbles and covered in plastic plants the preceding afternoon and collecting zebrafish embryos at 10 am in the morning, after the beginning of the light cycle. Zebrafish embryos were collected using plastic pipettes and placed in plastic jars before being transferred to the laboratory. Zebrafish embryos were cleaned 3-5 times in fresh 1X E3 embryo medium to remove organic matter. Next, zebrafish embryos were placed in a small tea strainer immersed for 3-4 minutes, with gentle swirling, in small petri dishes (⌀: 35 mm) filled with ~ 9 mL of medium in the following order: (i) bleaching medium, (ii) 1X E3 embryo medium, (iii) bleaching, (iv) 1X E3 embryo medium, and (v) 1X E3 embryo medium. Bleaching medium consisted in 0.004% bleach in 1X E3 embryo medium which was prepared by diluting 10-13% active sodium chloride in fresh 1X E3 embryo medium. Under a stereomicroscope (Zeiss), zebrafish embryos were then carefully moved to a petri dish (⌀: 90 mm) in fresh 1X E3 embryo medium. Viable embryos (at least 2-cells stage i.e. 0.75 hours post fertilisation — hpf — and no more than high stage i.e. 3.3 hpf, with no visible deformation nor chaotic cell division) zebrafish embryos were then carefully pipetted using a glass Pasteur pipette into 0.2 mL PCR wells pre-filled with the appropriate experimental medium for experimental treatments, ensuring to add no more than a few microliters (estimated < 5% of final volume) of additional medium.

For both protocols, embryos were incubated in 0.2 mL PCR wells placed in darkness in a thermocycler with a closed lid. Of note, constant darkness delays the normal development of zebrafish embryos (1) and our results have to be interpreted in this respect. For the repeated heat peaks treatment, every twenty-four hours of thermal stress protocol consisted of temperature fluctuations between 27, 29, 32, 29, and 27°C, with each temperature step being maintained for 15 min. This thermal stress mimicked +5°C temperature peaks over zebrafish optimal temperature reaching the sub lethal temperature of 32°C (2) and was in order to impose many heat peaks within a short timeframe to better understand the molecular mechanism of stress responses.

Depending on the endpoint as described below, zebrafish embryos experienced up to 19 heat peaks per 24 hours until they reached either 1 dpf (metabolomics, RNA-seq, phenotype data) or 4 dpf (cortisol, phenotype data). For the metabolomics data (endpoint 3), zebrafish embryos were exposed for 24 hours to a total of n = 13 heat peaks for 16 hours and 15 min followed by 7 hours and 45 min of recovery at 27°C, resulting from the experimental design of Feugere et al., 2021 (3). Of note, for the confirmatory experiment of endpoint 6, heat-stressed donors were exposed to a

constant 32°C in petri dishes. Embryos were incubated in E3 medium (i) free of any putative embryo metabolites ("fresh medium"), (ii) medium containing "stress metabolites" released by heat-stressed embryo donors, or (iii) medium containing "control metabolites" released by control embryo donors. Combining the temperature and metabolite factors yielded five treatments, where embryos were incubated as follows: in fresh medium at 27°C (control C), in fresh medium with thermal stress (TS), in medium containing stress metabolites at 27°C (SM), in medium containing stress metabolites with thermal stress (TS+SM), and in control metabolites at 27°C (CM). The four treatments C, TS, SM, and TS+SM together comprised a two-way factorial design (stress metabolites x thermal stress, Fig. 1B). Comparing SM and C to the additional control CM aimed to assess whether regular metabolites, only in higher concentrations are responsible for any observed effect of stress metabolites. Embryos in C and TS were metabolite donors for treatments CM and SM (where embryos were metabolite receivers). Conditioned medium was obtained from donors exposed to TS or C on each previous day to limit the degradation of metabolites. Conditioned media were renewed every 24 hours by pooling medium from donors' wells into a Falcon tube and immediately transferring 200 µL of the pooled conditioned medium into the receivers' wells.

Embryo medium

Embryo medium (4) was prepared by dissolving 34.8 g NaCl, 1.6 g KCl, 5.8 g $CaCl_2 \cdot 2H_2O$, and 9.78 g $MgCl_2 \cdot 6H_2O$ in ultrapure water to a final volume of 2 litres. The pH was adjusted to pH = 7.2 with NaOH and the solution was autoclaved and stored at 4°C. The working solution of 1X E3 embryo medium was prepared by 1:60 dilution of the 60X stock ultrapure water. For the metabolomics experiment, the embryo medium was prepared with autoclaved system water instead of ultrapure water.

Cortisol and HSP70 measurements

Embryo medium was renewed once a day by either fresh embryo medium (C and TS) or reused medium from donor embryos (CM and SM). Survival and hatching were monitored once a day at 11 am. At 4 dpf, viable zebrafish embryos were sampled and snap-frozen at -80°C until further processing for cortisol and HSP0 analyses. Experimental treatments were repeated several times using independent embryo clutches and treated embryos were distributed equally into biological replicates to limit any batch effects, until 60 embryos per biological replicate were obtained for each treatment (n = 3 samples per treatment, total of 180 embryos per treatment).

Cortisol was extracted from pooled samples using a method modified from Wilson et al. (5). Samples were thawed and 100 µL of ice-cold v/v solution of pre-autoclaved 1X PBS and molecular grade methanol were added to 1.5 mL polypropylene microcentrifuge tubes. Pooled embryo tissues were manually homogenised for approx. 30 sec using a pestle and briefly vortexed before incubation in ice for 15 min. Embryos were homogenised another 30 sec and sonicated on ice for 5 min at high intensity with 5 x 30 sec intervals of sonication time using the Bioruptor Sonicator (Diagenode). The homogenate was incubated 1 hr at 4°C on a rotary shaker with 10 rotations per min (rpm). The lysate was then centrifuged 5 min at 13,000 g at 4°C. Next, 20 µL of this supernatant were aliquoted to a separate tube and fourfold diluted in PBS for use in protein quantification using the Qubit™ Protein Assay Kit (Thermo Fisher Scientific) and for HSP70 analysis. At this step, zebrafish embryo samples were randomised for blind analysis in the ELISA

assay. The remaining volume of supernatant was transferred to a new sterile 1.5 mL microcentrifuge tube and was evaporated at room temperature under nitrogen flow until complete dryness after approx. 45-90 min. The pellet was reconstituted in 62 µL ice-cold ELISA assay reagent and resuspended by thorough vortexing before being stored at -80°C until cortisol quantification. Cortisol extraction recovery rates were estimated (90.9% ± 10.8%, n= 3) by spiking 100 µL of ice-cold v/v PBS/methanol with 10 µL of 100 pg/µL cortisol standard and following the same extraction method. The cortisol solution was obtained by a 10-fold dilution series from a 1 µg/µL cortisol standard stock solution containing 10.9 mg of H0396 (Sigma, 92 mg hydrocortisone per g powder) in 1 mL of molecular grade water. Cortisol levels were measured in duplicates using an Enzyme-linked Immunosorbent Assay (ELISA) Kit, following the manufacturer's recommendations (Salivary Cortisol Enzyme Immuno Assay Kit, 1-3002, Salimetrics, USA). Absorbance values were read at 450 nm with a reference at 490 nm using the BioTek ELX808 (NorthStart Scientific Ltd) plate reader. Cortisol concentrations in treated embryos samples and spiked samples were calculated from standard curve modelled by a four-parameter nonlinear regression using the online calculator mycurvefit.com (accessed 25/03/2021, $r^2 = 0.9999$, $p = 0.0001$, $F = 8{,}332$). Cortisol concentrations were normalised to the protein concentration of the embryo homogenate. Cortisol concentrations were transformed using the Lambert transform from the *bestNormalize* R package (6). Transformed values passed the residual normality and homoscedasticity assumptions and were compared across treatments using a one-way ANOVA. Post-hoc pairwise comparisons with false discovery rate p-value adjustment were computed using *emmeans* v1.7.2 (7) in R v4.0.2 (8).

HSP70 samples were obtained from the 20 µL supernatants saved before cortisol extraction. These lysates were diluted four-fold and processed for HSP70 analysis. Briefly, samples were incubated with Laemmli buffer, boiled for 10 min, and 15 µg proteins were resolved through a 10% SDS-PAGE gel. Proteins were transferred to nitrocellulose membranes and blotted for HSP70 using specific antibodies (#11565722, Fisher Scientific). Chemiluminescence signals were acquired using a ChemiDoc Imaging System (Bio-Rad). Membranes were stained by Ponceau S for loading control normalisation. Quantification of signals was done using Image J (U. S. National Institutes of Health, Bethesda, Maryland, USA, https://imagej.nih.gov/ij/). HSP70 intensities (validated in a second replicate) were normalised independently against the intensity of two separate Ponceau bands, and the average of these two ratios was then calculated. This was a blind analysis, where the experimenters and analysers did not know the identity of the samples. HSP70 protein levels were compared across treatments using a one-way ANOVA as values passed the residual normality and homoscedasticity assumptions.

RNA processing, sequencing, and data analysis

Embryos were pooled into groups of 20 embryos per sample in sterile nuclease-free 1.5 mL microcentrifuge tubes. Embryo pools were immediately snap-frozen at -80°C. Pooled samples were randomised prior to the RNA extraction for blind sample preparation and analysis. Total RNA was extracted using the TRIzol method following the manufacturer's recommended protocol followed by a DNAse I digestion step (#10792877, Invitrogen™ TURBO DNA-free™ Kit) which was followed by a sodium acetate cleanup. For this purpose, 0.1 mL of TRIzol reagent were added and embryos were homogenised using a plastic pestle for approx. 30 sec on ice. Further 0.4 mL of TRIzol were added to the samples, followed by another approx. 20 sec of homogenisation on ice

using a plastic pestle. To ensure that tissues were fully homogenised in TRIzol, samples were passed several times through a 200 µL pipette tip. Homogenates were centrifuged 10 min at 12,000 g to discard fat and debris, after which the supernatant was transferred to a new tube. TRIzol homogenates were incubated 5 min at room temperature to permit the dissociation of the nucleoprotein complex. Next, 0.2 mL of molecular grade chloroform:isoamyl alcohol 24:1 (#327155000, Acros Organics) were added and homogenates vigorously shaken, inverted, and vortexed, before being incubated 15 min at room temperature. Samples were centrifuged 15 min at 12,000 g at room temperature before the upper RNA-containing aqueous layer was transferred to a new tube. Next, 0.25 mL molecular grade isopropanol (99.5% #184130010, Acros Organics) were added, and samples were mixed by several inversions before being left to incubate 10 min at room temperature. Samples were centrifuged 10 min at 12,000 g at room temperature (to prevent salt precipitation) to pellet the RNA. The supernatant was discarded. RNA pellets were cleaned three times in ethanol by resuspending in 0.5 mL molecular grade ice-cold 75% ethanol and were centrifuging 5 min at 10,000 g at 4°C before discarding the ethanol phase. Excess ethanol was removed using a pipettor and evaporated by air-drying at room temperature for approx. 3 min and at 55°C on a block heater for 2 min. Next, RNA pellets were resuspended in 70 µL molecular grade water and heated for 5 min at 55°C with regular vortexing. DNA was removed by a routine DNAse I treatments (#10792877, Invitrogen™ TURBO DNA-free™ Kit). For this purpose, 0.1 volume of 10X Turbo DNAse Buffer and 1 µL DNAse enzyme were added to the RNA and gently mixed before 20 min incubation at room temperature. 0.1 volume of DNAse inactivation reagent was then added before 5 min incubation at room temperature with regular mixing by flicking the tubes. Samples were centrifuged for 90 sec at 10,000 g at room temperature. Next, 80 µL of DNA-free RNA solutions were transferred to a new tube and samples placed on ice. RNA samples were purified using sodium acetate protocol modified from Walker and Lorsch (2013) (9) to remove impurities such as phenol and protein contamination. 3M sodium acetate was prepared by adding 12.3 g of sodium acetate anhydrous (#BP333-500, Fisher scientific) in 7.6 mL glacial acetic acid (#A/0360/PB15, Fisher scientific, to adjust the pH to 5.2) and completing the volume to 50 mL in molecular grade water before autoclaving the solution. Next, 0.1 volume of ice-cold 3M sodium acetate and 2.5 volumes of ice-cold molecular grade absolute ethanol were added to RNA samples. Samples were left to incubate at -20°C for 2 hours and RNA was pelleted at 12,000 g for 15 min at 4°C. Next, the supernatant was carefully removed, and the RNA pellet washed three times by adding 200 µL ice-cold molecular grade 75% ethanol, letting the pellet to soak for 2 min (in ice) before centrifuging 2 min at 12,000 g at 4°C, and removing the ethanol supernatant. Purified RNA was resuspended in 75 µL molecular grade water by heating 2 min at 55°C on a block heater. Aliquots of 10 µL were taken for quality and quantity assessment, whilst the remaining 65 µL were stored at 80°C before being sent in dry-ice to Edinburgh Genomics for RNA-sequencing. RNA purity ratios were assessed using the NanoDrop 1000 Spectrophotometer. All RNA samples contained 65 µL with ≥ 1,200 ng RNA, and sufficient purity as shown by 260/280 and 260/230 ratios respectively > 1.9 and > 1.5.

Total RNA samples were further processed by Edinburgh Genomics to generate cDNA libraries using the TruSeq stranded mRNA kit. cDNA libraries were cleaned when necessary and samples were loaded onto two flow cell lanes and sequenced by Illumina NovaSeq 50PE sequencer. Raw read files were processed to remove Illumina adapters, polyG and polyX tails using *fastp* v0.23.1 (10), with deduplication disallowed (--adapter_fasta adapters.fasta --dup_calc_accuracy 3 --trim_poly_g --trim_poly_x). The genome indices were generated with *STAR* default options.

Reads were mapped to the genome using *STAR* v2.6.1 (11) and counted by genes (--runMode alignReads --quantMode TranscriptomeSAM GeneCounts --outSAMtype BAM SortedByCoordinate --outSAMunmapped None --outSAMattributes Standard) to generate read count table outputs.

Total RNA samples assessed for integrity (assessed by electrophoresis in 1.2% agarose gels) and concentration (measured by Qubit Broad Range kit, Invitrogen, and QubitTM fluorometer 3.0) were analysed at the Edinburgh Genomics facility. Libraries were prepared with the TrueSeq stranded mRNA kit and sequenced by Illumina NovaSeq 50PE sequencing. The RNA-seq analysis for differential gene expression was performed on the VIPER High-Performance Computing facility hosted at the University of Hull. Read quality was assessed using *FASTQC* v0.11.9 (https://www.bioinformatics.babraham.ac.uk/projects/fastqc/). Reads were filtered and trimmed using *fastp* v0.23.1 (10) and reassessed for quality control using *fastp* and FASTQC. The absence of PCR-biased duplication was assessed using the R package *DupRadar* v1.18.0 (12). Next, the splice-aware mapper *STAR* v2.6.1 (11) was used to align the reads (see summary statistics in Tables S10-S12) to the zebrafish genome (primary genome assembly GRCz11.104) in order to obtain read counts. Differential gene expression analysis was performed using *DESeq2* v1.28.1 (13) in R v4.0.2 (8) using *Bioconductor* v3.11 (14). Gene expression was analysed using the treatments as a single factor in the matrix design, and performing pairwise comparisons (SM, TS, and TS+SM versus control C) were retrieved from the results function with the "cooksCutoff" option disabled since there was no evidence of extreme outlier. The Principal Component Analysis was represented after regularised logarithmic (rlog) transformation using the assay (from *SummarizedExperiment* v1.18.2 (15)) and prcomp functions. The differentially expressed genes (DEGs) were visualized as volcano plots drawn using the *EnhancedVolcano* package v1.6.0 (16). Gene names were converted using the *biomaRt* R package v2.44.4 (17, 18).

Differentially expressed genes were analysed by biological process GO terms using *topGO* v2.40.0 (19) to investigate the effect of TS and SM respectively on genes related to heat stress (GO:0009408) and chemosensory perception (GO:0007606) with GO term-wide false discovery rate p-value adjustment. We reasoned that temperature stress would induce whole-body transcriptomic changes, hence multiple testing-adjusted p-values ($p\text{-adj} < 0.05$, controlling for type I error i.e. false positives) were used to subset genes with evidence for altered expression in the functional enrichments. On the other hand, based on previous transcriptomic studies on chemical cues (20–22), we expected that stress metabolites would activate a more local transcriptomic response related to the sensory system and downstream pathways, which we decided to explore via a more relaxed explorative approach through using unadjusted raw p-values ($p < 0.01$).

The functional enrichment for GO terms (BP: biological processes), Kyoto Encyclopedia of Genes and Genomes (KEGG) (23), and *Reactome* pathways were performed using the *GOstat* v2.54.0 (24), *clusterProfiler* v3.16.1 (25), and *ReactomePA* 1.32.0 (26) R packages, respectively, and visualised for significant ($p < 0.05$) terms having gene counts ≥ 2 (but keeping all significant terms regardless of gene counts to interpret the functional enrichment). Relevant information about specific DEGs was retrieved from the Zebrafish Information Network (27). In addition, when annotating the SM-responsive DEGs in the ZFIN database, we noticed that several of them were previously mentioned in a publication by Elkon et al., 2015 (28) which we used for screening genes

expressed both in SM and hair cells of the lateral line system of 5-dpf zebrafish embryos, in light of the possible role of the lateral line hair cells in detecting chemical cues (29).

Metabolomics

LC-MS/MS

170 µL of medium per embryo were pooled for 60 embryos (20 embryos from 3 clutches) and filtered (Costar® Spin-X®, 0.45 µm Pore CA Membrane) to yield 10.2 mL medium for each CM, SM, and the blank. Orbitrap LC-MS/MS analysis and raw data preprocessing were performed at the Metabolomics & Proteomics facility of the University of York. Each embryo medium (SM, CM, or blank) was split into three technical replicates for a total of 9 runs for each ionisation mode (positive and negative). Two microlitres of provided samples were injected onto a Waters HSS T3 100 mm x 2.1 mm (1.8 µm particle) column, using an Acquity I-Class UPLC system. Compounds were eluted over a 9 min gradient of water/acetonitrile/ 0.1 % (v/v) acetic acid at column temp of 40°C and a flow rate of 0.5 mL/min. The column was conditioned with six runs of pooled samples before acquiring usable data. Each sample was injected twice, for separate positive and negative ESI mode analysis on a Thermo Tribrid Fusion Orbitrap MS system.

Raw MS data preprocessing

Data was collected between 0.5 and 8.5 min, in data dependent MS2 mode. This collected high-resolution MS1 data (60000 FWHM at m/z 200), and low resolution MS2 scans in both CID and HCD modes. The MS1 data (approx. 2 scans/s) was used for feature picking and quantification. Acquired Thermo .raw files were converted to 32-bit precision centroided .mzML files using MSConvert version 3.0.20023-2701dc40b. These were then processed using bespoke R scripts (R 4.0.3 64 bit in a Linux environment). Briefly, features were selected using the xcmsSet() function from the *xcms* package (v 3.12.0; 30), with the following parameters: method = 'centWaveWithPredictedIsotopeROIs', ppm = 5, snthresh = 10, peakwidth = c(3, 30), prefilter = c(3, 1000), integrate = 2, mzdiff = -0.1. Features were then grouped, and missing values imputed using derived xcms functions. Feature relationships were identified using the *CAMERA* package v1.33.3 (28), and candidate formulae calculated using the *rcdk* package v3.5.0 (32, 33). Where appropriate, MS2 spectra were extracted using bespoke scripts, and data tables and plots generated using adapted R functions. Using bespoke R scripts, the annotated features were identified and assigned a unique identifier (hereafter, “masstag”) informing the monoisotopic mass m/z and retention time in seconds. There were respectively n = 3,345 and n = 2,238 masstags in the positive and negative ionisation modes, yielding a grand total of n = 5,583 masstags.

Compound annotation, filtering, and biomarker identification

Next, masstags were automatically annotated using available libraries. First, masstags were automatically matched against a custom list of n = 30 potential candidate compound references (**Table S2**). Second, the web-based platform *MetaboAnalyst* v5.0 (34) was used for automatic matching of masstags to known compounds using the Functional Analysis of MS peaks module (https://www.metaboanalyst.ca/MetaboAnalyst/upload/PeakUploadView.xhtml). For each negative and positive mode of the nine samples from the three media (CM, SM, and blank), raw peak intensity data was entered with retention times in seconds and mass tolerance of 5 ppm. The data was left unfiltered (recommended for n features < 5,000), sample normalised by median

(negative mode) or sum (positive mode), cube-root or (negative mode) transformed, and auto-scaled (i.e. mean-centered and divided by the standard deviation of each variable). Matched compounds were retrieved from the mummichog v2.0 algorithm with a custom currency metabolite exclusion list (water, proton, oxygen, NADPH, NADP, NADH, NAD, carbon dioxide) whilst including all available adducts in positive and negative modes. In order to maximise the compound matching, the mummichog algorithm was repeated using all available libraries from the following available animal species: the zebrafish *Danio rerio* (MetaFishNet (35) or MFN library covering KEGG zebrafish model, human BiGG and Edinburgh Models) for fish; the human *Homo sapiens* (MTF library covering KEGG, BIGG, and Edinburgh Model databases); the mouse *Mus musculus* (KEGG and BioCyc), the rat *Rattus norvegicus* (KEGG), the cow *Bos taurus* (KEGG) for mammals; the chicken *Gallus gallus* (KEGG) for birds; the fruit fly *Drosophila melanogaster* (KEGG, BioCyc) for insects; and the nematode *Caenorhabditis elegans* (KEGG). After filtering duplicates, this yielded a grand total of 217 and 281 unique matched compounds from the mummichog algorithm in the negative and positive modes, respectively. To exclude the masstags predominantly present in the blank, a threshold cutoff was defined as in Equation 1.

$$Cutoff = 2 \times (\text{ mean } Intensity_{blank} + 3 \times SD\ Intensity_{blank}) \qquad \textbf{Equation 1.}$$

Masstag intensities were blank corrected (BC, equation 2) and their intensity expressed as blank corrected percent total relative to the sum of masstag areas per sample (BCP, Equation 3).

$$BC_{Masstag} = Area_{Masstag}^{Sample} - Cutoff_{Masstag} \qquad \textbf{Equation 2.}$$

$$BCP = 100 \times (BC_{Masstag} / \sum_{i=1}^{per\ sample} BC) \qquad \textbf{Equation 3.}$$

Because our analysis only used one true biological replicate (pooled medium sample from 60 embryos) but with technical replicates, we aimed to avoid pseudoreplication bias and therefore did not use statistical analysis of significance between CM and SM. Instead, the data was first filtered to account for signal-to-noise ratio by only selecting masstags with intensities over the threshold cutoff in at least three samples out of six technical run replicates (n = 3 in CM, n = 3 in SM). This narrowed the potential biomarkers of SM and CM media to n = 89 unique masstags from the positive and negative modes altogether. Second, the biomarkers were assigned to either SM or CM based on which group their average blank corrected percent total intensity was highest. Third, the suitability of biomarkers B for either group was measured as the absolute difference between the intensities in CM and SM media (Equation 4), with higher B values indicative of suitable biomarker for either condition.

$$B = |CM - SM| \qquad \textbf{Equation 4.}$$

To complement the aforementioned automatic annotation of all 5,583 masstags, the identity of the masstags was manually matched with available online databases using their m/z masses. The two main databases, for which all available positive and negative ion adducts, and a tolerance threshold of ± 0.0005 m/z were used, were (i) the Direct Infusion MEtabolite database (DIMEdb, (36) and (ii) the Metabolomics Workbench (The Metabolomics Workbench, https://www.metabolomicsworkbench.org/ with three options: using the reference set of metabolite species RefMet; the Metabolomics Workbench Metabolite database with a m/z value; and the untargeted metabolite search option). Additional manual annotations using the narrowest

available mass tolerance range were retrieved from other databases including the metabolomic Fiehn lab's LipidBlast database (37), The LIPID MAPS® Lipidomics Gateway (https://www.lipidmaps.org/), the National Institute of Standards and Technologies (NIST) Chemistry Book, UniProt (38), MassBank Europe (https://massbank.eu/MassBank/), mzCloud (https://www.mzcloud.org/), as well as a general screening of the available literature and supplemental data wherein. This manual screening of > 5,000 entries identified approx. 2,500 possible hits (including within and cross-database duplicates). Each potential hit was assigned a compound name and its identifier from common chemical registries (of which PubChem, Human Metabolome DataBase or HMDB, and KEGG). Next, the literature and publicly available metabolome information corresponding to each possible hit was thoroughly screened (HMDB, KEGG, Food database, PubChem-associated literature, clinical trials, and patents; literature search; ChEBI, MetaCyc, KNApSAcK) to enhance the compound annotation by removing potential hits not likely to be found in pre-hatching zebrafish embryos exposed to our experimental treatments. For this purpose, the exclusion criterions were: no biological records, fungi, bacterial or plant metabolites, xenobiotic metabolites, or industrial or pharmaceutical use. On the other hand, the inclusion criterions were that the potential hits were previously found in metazoan species, general metabolites, or could not be disregarded using the exclusion criterions. It should be acknowledged that although this filtering is slightly prone to subjectivity, it mainly ought to limit the risks of associating masstags to unlikely compound matches.

Functional Enrichment

Subsequently, retained possible hits were annotated with their IUPAC International Chemical Identifiers (InChI or InChI Key) which were used for automatic structure-based ChemOnt chemical taxonomy classification using the ClassyFire algorithm (39; available at http://classyfire.wishartlab.com/queries/new or https://cfb.fiehnlab.ucdavis.edu/, accessed July 2021). We reasoned that because one cannot assign a unique metabolite per masstag amongst several isomer candidates, one should use only one compound per level of classification in order to limit the redundancy of the functional enrichment. Therefore, where relevant (i.e. if there are possible hits and different chemicals sharing the same classification), one compound per subclass (or class and superclass when compounds are not classified to the subclass level) per masstag that had either a KEGG or HMDB identifier was selected as a representative compound for the functional enrichment. This approach aimed to limit the redundancy of subclasses in the functional enrichment and prevent bias towards subclasses for masstags that match several known compounds. This yielded 125 representative annotated compounds used for both chemical structure (sub-class) and pathway (KEGG) functional enrichment using the *MetaboAnalystR* v3.0 (40) R package in *Rstudio*. The functional enrichments were performed for SM- and CM-specific filtered metabolites separately, with Holm's p-value correction for multiple testing.

Multi-omic integrative analysis

Next, the transcriptome and metabolome data were integrated into two joint analyses. First, the Joint Pathway from MetaboAnalyst was used to explore the enriched KEGG pathways. Only the representative hits of the stress medium were used in the joint pathway analysis to avoid an artificial bias as masstags could have several hits with the same ChemOnt classification. The Joint Pathway analysis looked for the enriched KEGG pathways using a hypergeometric test using

MetaboAnalyst v.5.0 (34, 41), (https://dev.metaboanalyst.ca/MetaboAnalyst/upload/JointUploadView.xhtml, accessed February 2022 — options were set to: organism: *Danio rerio*; Metabolomics type: “targeted (compound list); ID type: “official gene symbol” and “compound name”; topology measure: “degree centrality”; integration method: “combine queries”, pathway database: “metabolic pathways”).

Second, the compound-protein interactions (CPI) were sought in the STITCH (42, 43) database using Cytoscape v3.9.0 (44). Out of the 202 unique possible metabolites in SM, several compounds (proteins, lipids, and polypeptides) would not be found in STITCH and were removed, leaving 46 compounds that were manually searched in the online version of STITCH (http://stitch.embl.de/, accessed February 2022). These filtered n = 29 compounds that existed in the STITCH database were used for the CPI analysis. We reasoned that the significant genes in TS may lead to the synthesis and release of stress metabolites in the stress medium, and that the stress metabolites would initiate a transcriptome response in embryos of the SM treatment. Therefore, both the joint pathway and CPI analyses were performed twice by combining the candidate metabolites with genes of either SM or TS. To optimise the readability of the networks and to account for the number of genes, the CPI analyses for TS (n = 126 identifiers) and SM (n = 94 identifiers) were performed at confidence score cutoff of 0.7 and 0.4, respectively, with the following options: network type: full STRING network; no additional interactors, no singletons.

LAMP data

Three sets of 20 embryos each obtained from outcrossing pet store line (PET) zebrafish maintained at the University of Hull since 2018 were exposed in groups in petri dishes for 24 hrs to either fresh E3 medium or SM (n = 60 embryos per sample, 250 µL medium per embryo). Stress metabolites were obtained from donors experiencing constant thermal stress of 32°C from 0 to 1 dpf. Total RNA was extracted from 4 such pools per treatment as previously described. Fluorometric LAMP (Loop-mediated isothermal amplification reactions) were performed according to manufacturer’s protocol (NEB #M1708S) with primers (Dataset S1) designed for selected RNA-seq DEGs: chitin synthase 1 (*chs1*), lactate dehydrogenase A4 (*ldha*), olfactory receptor class A related 3 (*ora3*), otoferlin a (*otofa*), proteoglycan 4a (*prg4a*), and toll-like receptor 18 (*tlr18*). Reactions were run in technical triplicate on a StepOne qPCR machine. Gene expression was quantified using the $-\Delta\Delta C_T$ method (45) representing the time to reach the threshold (C_T with each cycle lasting 1 min) normalised to *β-Actin* as housekeeping gene and scaled to control values. Gene expressions were compared using Student’s t-tests.

Phenotypic analysis

Embryos were collected around 10 am, cleaned through bleaching, and selected under the stereomicroscope. 1-dpf larvae were used for light-induced startle responses videoed under the stereomicroscope during 30-s videos. Embryos were then gently manually dechorionated under the stereomicroscope and individually imaged in lateral view on a microscope slide with a micrometre scale, before being placed in new individual wells pre-filled with 200 µL of experimental medium. Embryos were then individually exposed to treatments within their chorion from 0 dpf to 1 dpf. After 24 hours, embryos were removed from their individual wells and placed in watch glasses with a small amount of medium. Experimental conditions were maintained until

4 dpf. At 2 and 3 dpf, 180 µL of medium were replaced with either fresh or conditioned media. All embryos were monitored daily for hatching and survival.
At 4 dpf, larvae were individually transferred into flat-bottom wells of 35 mm diameter pre-filled with 3 mL fresh embryo medium pre-warmed at 27°C. The starting temperature was measured using a small portable thermometer. A Canon 1200D camera was used to record the swimming behaviour. The 6-well plate was positioned at the centre of a photobox. Embryos were allowed to settle for 30 seconds after being placed in their swimming wells before the video started. The swimming assay consisted of touching the head of the embryo with the loop of an inoculating needle to record the touch-evoked swimming behaviour. One escape response was triggered every 20 seconds with three repeats per embryo to measure their stamina. Once all videos were completed, the 4-dpf (~ 100 hpf) were imaged on a microscope slide with a micrometre scale.

Images and videos automatically bulk renamed and randomised (using Bulk Rename Utility v3.4). Imaging analysis was completed using Image J v1.53e (46) after image randomisation for screening regions of interest (Fig. S1B) in 1- and 4-dpf zebrafish individuals. For 1-dpf embryo, these were: shortest embryo length (SEL, shortest embryo length from head to tail), longest embryo length (LEL, segmented line in the anterior-posterior axis from the epiphysis in the middle of the head to the tail), yolk ball length (YBL) and area (YBA), yolk extension length (YEL) and area (YEA), tail width (TW, cross section of the caudal fin in dorsal-ventral axis where the notochord ends), eye length (EL) and area (EA), dorso-ventral length (DVL, longest dorso-ventral cross section in the yolk ball region), and whole-body area (WBA). In addition, several staging indexes were used to best estimate the developmental stage of the embryos, including the head-trunk angle (HTA, adjacent angle to the angle formed between a line crossing through the middle of the eye and the otic vesicle and the line parallel to the notochord between the 5th and 10th somites), the otic vesicle length (OVL, i.e. how many more otic vesicles would fit between the otic vesicle and the eyes), yolk extension-to-yolk ball ratio (YE/YB). Embryo staging was also guided by binary coding for the presence of features such as the eye primordium (≥ 19.5 hpf), two clear otoliths (≥ 22 hpf), the hatching gland (≥ 22 hpf), the cerebellum formation (≥ 25 hpf) and its migration towards the head (≥ 31 hpf), or the tail strengthening (≥ 31 hpf). In 4 dpf embryos, the EL, SEL, DVL, TW, and WBA were measured.

Video of 1-dpf startle responses were analysed using Danioscope (Noldus). Video analysis at 4 dpf was completed using KINOVEA v0.9.5 (47) after video randomisation. The total distance and number of burst events were measured following each of three stimuli for the entire video. Second, speed and acceleration following the 1st (first) and 3rd (last) stimuli were monitored at each frame for the first complete burst defined as when embryos reached full immobility (0 m/s) for at least two consecutive frames regardless of subsequent bursts. Comparing responses between the first and last touches allowed us to calculate the delta total distance to estimate the stamina of embryos. However, since Welch's adjusted t-tests showed that there were no significant differences between the 1st and 3rd stimuli for the mean acceleration ($t = -0.01$, $df = 284.08$, p-value = 0.99) and mean speed ($t = 0.99$, $df = 313.8$, p-value = 0.32), these were averaged across the first and last stimuli.

All phenotypic data analysis was conducted in R v4.0.2 (8) using *Rstudio* v1.3.1056 (48) with *ggplot2* v3.3.5 (49) for graphs. Outliers were excluded using repeated Grubbs tests for one outlier from *outliers* v0.14 (50). For each response variable, models were fitted to estimate the effects of (i) thermal stress and stress metabolites across the two-way factorial design (C, SM, TS, TS+SM),

followed by pairwise post-hoc comparisons relative to control C, and (ii) of media by post-hoc comparing CM to C and SM. For each analysis, covariates were included when there was evidence of their significance for the model according to the Akaike Information Criterion of the *aictab* function from the *AICcmodavg* v2.3-1 R package (51) to sort possible models by delta AICc, stepAIC from *MASS* v7.3-51.6 (52) to estimate the best model from the full model, and *drop1* function from the *stats* R package to estimate whether any terms could be dropped. Since embryos were not fixed in agarose for imaging, approx. 30% of the images could not capture the otic vesicle which prevented the measurement of several variables of interest. Therefore, only complete cases (n = 114 out of 157 embryos) were retained for a multivariate analysis using a PERMANOVA using the *adonis* function from the *vegan* v2.5-7 R package (53). Morphology data was represented by a principal component analysis (PCA) of treatment groups using the *ggbiplot* v0.55 (54) and *prcomp* R functions. All pairwise comparisons were corrected by Tukey's method for false discovery rates. If necessary, data were normalised using *BestNormalize* v1.8.2 (6). The homoscedasticity and normality of model residuals were assessed using Shapiro-Wilks' and Studentized Breusch-Pagan tests from *lmtest* v0.9-38 (55), respectively. Continuous data of the two-way factorial design were analysed by ANOVAs or Scheirer-Ray-Hare tests from *rcompanion* v2.4.1 (56) for parametric and non-parametric data, respectively. Binary data were analysed using generalised linear models (GLMs) with binomial distribution. Burst count data was analysed using GLMs with negative binomial distribution from *MASS* (52), as this distribution best fitted the data according to *fitdistrplus* v1.1-6 (57), combined with a two-group equal density test from *sm* v2.2-7.7 (58). Pairwise comparisons were retrieved from models (ANOVAs and GLMs) using *emmeans* v1.7.2 (7), Wilcoxon-Mann-Whitney tests (non-parametric data), or *pairwiseAdonis* v0.4 (59) (PERMANOVAs). Effect sizes of model terms were represented by Cohen's |d| values, approximated as $d = 2 \times f$ (60) from Cohen's f values from *effectsize* v0.6.0.1 (61) and interpreted according to Sawilowsky (62) as tiny ($|d| < 0.1$), very small ($|d| > 0.1$), small ($|d| > 0.2$), medium ($|d| > 0.5$), large ($|d| > 0.8$), very large ($|d| > 1.20$), and huge ($|d| > 2.0$). Phenotypic data were interpreted using $p < 0.05$ for marginal evidence and $p < 0.01$ for strong evidence, whilst considering $p \leq 0.08$ for trends with weak evidence.

<u>Supplementary Results</u>

Transcriptomic response to heat stress

The principal component analysis of RNA-seq data did not show evidence that samples clustered by treatments across either of the first two variance axes (PC1 = 51% and PC2 = 16% of variance, respectively, Fig. S3). Analysis of the gene subset for the Biological Process GO term "response to heat" (GO:0009408) provided strong evidence that there was a more than three-fold overexpression of four genes related to the heat shock response, namely heat shock protein 90, alpha (cytosolic), class A member 1, tandem duplicate 20 (*hsp90aa1.2*), heat shock cognate 70-kDa protein, tandem duplicate 3 *(hsp70.3*), heat shock protein family A (Hsp70) member 8b (*hspa8b*), and the heat shock cognate 70 (*hsc70*). These transcriptional changes within members of the heat shock response explained why "cellular response to heat" (GO:0034605) as well as "cellular response to heat stress" and "regulation of HSF1-mediated heat shock response" were amongst the most significantly enriched functions in this comparison (Fig. 2A, 2D, Dataset S1). Protein refolding processes (e.g. GO:0042026; GO:0034620) were also significantly enriched and included *hsp90aa1.2*, *hsp70.3*, *hspa8b*, and *hsc70*. Furthermore, the functional enrichment highlighted that the top thirty gene ontology terms with the strongest evidence (all p-adj < 0.009)

of heat-induced whole-body transcriptome response were mainly ascribed to three biological processes: sensory perception, development, and metabolism (Fig. 2D, S4). The most significantly enriched biological processes were associated with the eyes as evidenced by the terms "lens development in camera-type eye" (GO:0002088) and "*visual perception*" (GO:0007601). These GO terms mostly included a total of six genes linked to the lens and cornea proteins crystallin alpha (*cryba1*, *cryba1l1*, *cryba2a/b*, and *crybb1l1/2*) and eight to crystallin gamma (*crygm2d8/10/13/17/18/20*, *crygmx*, and *crygmxl2*). In addition to the eyes, other development-related functions included GO terms such as "multicellular organism development" (GO:0007275), "animal organ development" (GO:0048413), "developmental process" (GO:0032502), "somite specification" (GO:0001757), and "muscle fibre development" (GO:0048747). The Reactome database enrichment also indicated an alteration of muscle-related gene expression with the term "striated muscle contraction".

Therefore, our data suggested that heat stress significantly impacted the morphogenesis of somites and muscular structures during embryogenesis. Of note, all six genes related to somite development (GO:0001757, GO:0061053, and GO:0035282) terms were downregulated in thermal stress. These included the mesoderm posterior ab (*mespab*), as well as four genes also found in the "negative regulation of transcription by RNA polymerase II" term (GO:0000122), namely hairy-related 1 (*her1*), hairy and enhancer of split related-7 (*her7*), ripply transcriptional repressor 2 (*ripply2*), and T-box transcription factor 6 (*tbx6*). Further, muscle development in heat stressed embryos was associated with elevated transcript levels of three paralogues of actinidin (*and1/2/3*), as well as myosin, heavy polypeptide 2, fast muscle specific (*myhz2*), but also three myozenin genes (*myoz1a/1b/3a*). On the other hand, there was evidence that the gene expression of myogenin (*myog*) and myosin 7 (*CU633479.2*) were significantly inhibited by high temperatures. The third major GO term category with evident significant functional enrichment covered several metabolism and energy pathways such as the regulation of adenosine diphosphate (ADP, GO:004603), triphosphate (ATP, GO:0046034), and glycolytic (GO:0006096) metabolic processes. Notably, these terms evidenced the heat-induced activation of several transcripts of enolase (*eno1a* and *eno3*), glyceraldehyde-3-phosphate dehydrogenase, spermatogenic (*gapdhs*) but also genes with a kinase activity such as phosphoglycerate kinase 1 (*pgk1*) and pyruvate kinase M1/2b (*pkmb*). Additional functional enrichment for Reactome (Fig. S4A) and KEGG (Fig. S4B) pathways confirmed the high temperature-induced alteration of the metabolism of ADP/ATP, glucose (e.g. terms "gluconeogenesis/glycolysis", "pentose phosphate metabolism"), "amino acid" as well as "pyruvate metabolism". In summary, this shows that heat stress induces transcriptomic changes in embryonic development (of the eyes, muscles, and somites), the metabolism of sugars, amino acids, purine, and energy intermediates (ATP and pyruvate), and in the heat stress response.

Transcriptomic response to Stress Metabolites

For the genes differentially regulated by SM, the cytokine production GO terms (e.g. GO:0001816) were associated with the genes *anxa1a* (*annexin A1a*) and *scamp5a* (*secretory carrier membrane protein 5a*). The GO term with the highest gene count (n = 8) was "intracellular signal transduction" (GO:0035556) and included the most upregulated gene *si:ch211-214b16.2 (NOD2* ortholog). Moreover, the genes *fthl28* (*ferritin, heavy polypeptide-like 28*) and *tlr18* (*toll-like receptor 18*) led to the enrichment of the cell death-related Reactome terms "ferroptosis" and "necroptosis". Regardless of the database, there was evidence that one set of genes initiated the

pathways of glucosamine and glycosaminoglycans (e.g. “chitin biosynthetic process” — GO:0006031, “keratinization”, and “keratan sulfate/keratin metabolism”). These genes included the upregulated keratin genes (*krt97/17*) and chitin synthase 1 (*chs1*) as well as the downregulated *carbohydrate sulfotransferase 2b* (*chst2b*). Further, several other keratin genes (*krt92/222*) and cytokeratin *cyt1* (type I cytokeratin, enveloping layer) were also upregulated in SM. Several terms associated with anion and lipid transport were also enriched such as “phospholipid transport” (GO:0015914), “lipid localisation” (GO:0010876), and “anion transport” (GO:0006820) which included genes such as *anxa1a* (annexin 1a), *best1* (bestrophin 1), *bscl2l* (BSCL2 lipid droplet biogenesis associated, seipin, like), *pitpnc1b* (phosphatidylinositol transfer protein cytoplasmic 1b), and *xkr8.2* (XK related 8, tandem duplicate 2).

Phenotypic data: behaviour, development, hatching, and survival

This showed that the percentage of moving embryos did neither vary with heat ($z = -1.28$, $p = 0.2011$) nor with stress metabolites ($z = 1.02$, $p = 0.3088$). There was nevertheless a decrease in moving embryo % between CM (57.1%) and SM (34.7%, $z = -2.2098$, $p = 0.0542$, Fig. S10F). Because over half (52.4%) of the embryos were immobile, the burst count per minute was re-analysed only for active embryos, showing an effect of TS and TS+SM (Fig. 5B, Tables S7-S8). At 4 dpf, embryos incubated in CM swam longer distances than those in SM ($t = -2.22$, $p = 0.058$, Fig. 11D).

We also recorded both mortality (Fig. S10C) and hatching (Fig. S10D) in over 1,000 embryos across more than 30 batches. There were no significant differences in mortality across treatments (analysis of deviance $p = 0.0951$) with an average survival of 92% which confirmed that all experimental protocols were sublethal. Next, there were no significant differences in hatching percentages between C and SM ($z = 0.3765$, $p = 0.7066$). On the other hand, hatching was significantly higher in TS (43%) compared to C (25%, $z = -5.79$, $p < 0.0001$), but also high in CM (63%) compared to C ($z = 9.52$, $p < 0.0001$) and SM (33%, $z = 9.03$, $p < 0.0001$).

The first axis (PCA1 = 34.1% of variance) discriminated treatments under thermal stress (TS and TS+SM) from treatments at control temperature (Fig. 5A, Table S4). Testing individual variables confirmed that thermal stress significantly increased the length and surfaces of whole-body embryos (Fig. 5C), but also their eye, and yolk elongation, but decreased the head-trunk angle and otic vesicle length (Fig. 5A), as well as the percentage of defects (Fig. S10A). Thermal stress also significantly accelerated (median stage: 31 hpf, $H = 50.36$, $p < 0.0001$, very large effect size = 1.47) the growth of embryos compared to control C (median stage: 25 hpf) within the first 24 hours of development. Therefore, heat-stressed embryos reached the pharyngula stage earlier ($z = -2.67$, $p = 0.0076$, Fig. S10B). After statistically accounting for the large effect size of batch ($d = 1.10$, $t = 38.33$, $p < 0.0001$) and its interaction with heat ($t = 5.51$, $p = 0.0206$), there was a marginal increase in length with thermal stress in one batch of 4-dpf larvae. This led to embryos in TS+SM having increased 4-dpf body lengths compared to control embryos ($t = 2.41$, $P = 0.0520$, Fig. 5D). There was also a significant decrease in ΔSEL in TS compared to C ($t = -3.34$, $p = 0.0034$). ΔSEL was however similar in CM compared to SM ($p = 0.3133$) and C ($p = 0.9307$). The pattern of slower growth with high temperature between 1 and 4 dpf was confirmed for the eye length increment (ΔEL) which tended to marginally — albeit not significantly ($t = 3.29$, $p = 0.0724$) and mainly in one batch — decrease with thermal stress (Fig. S11A).

Supplementary Figures

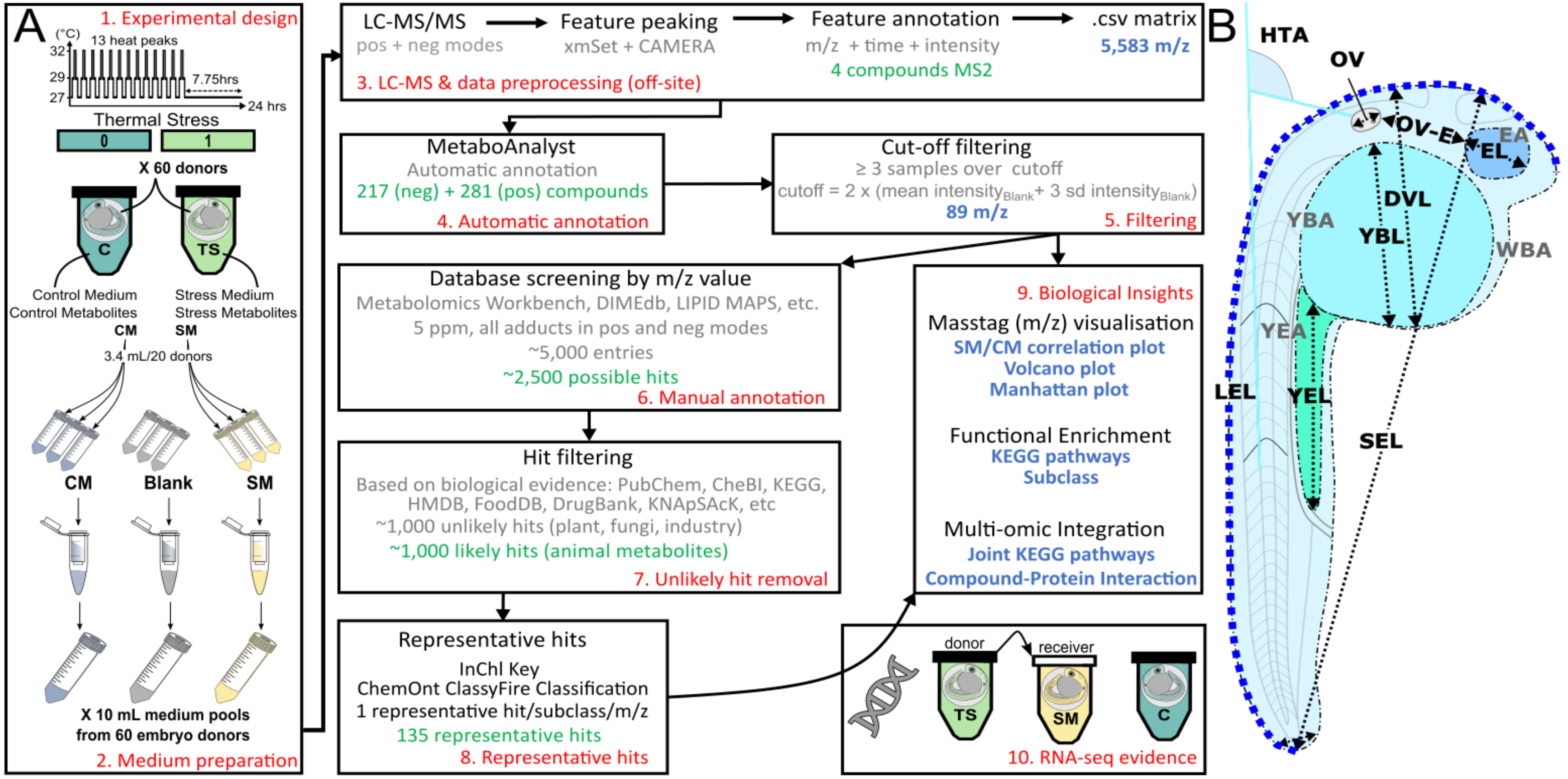

Figure S1. A) Schematics of multi-omic analysis. Red font: steps 1 to 10 of multi-omic analysis. Green font: compound identification. Blue font: masstag (m/z) data. Multi-omic analyses integrate data from donors (TS) and receivers (SM) of stress metabolites using RNA-seq and metabolomics. **B) Measurements of zebrafish embryos quantified in Image J.** Lengths (dashed lines) and areas (dot-dashed lines) are shown in black and grey font, respectively. LEL: longest embryo length, SEL: shortest embryo length, YEL: yolk extension length, YBL: yolk ball length, OV: otic vesicle, OV-E: otic vesicle to eye length. OVL is the ratio OV-E/OV. EL: eye length, DVL: dorsal-ventral length, YEA: Yolk extension area, YBL: yolk ball area, WBA: whole-body area. HTA is the head-trunk angle formed between the cyan blue lines. Criteria are based on Kimmel et al. 1995 (63)**.**

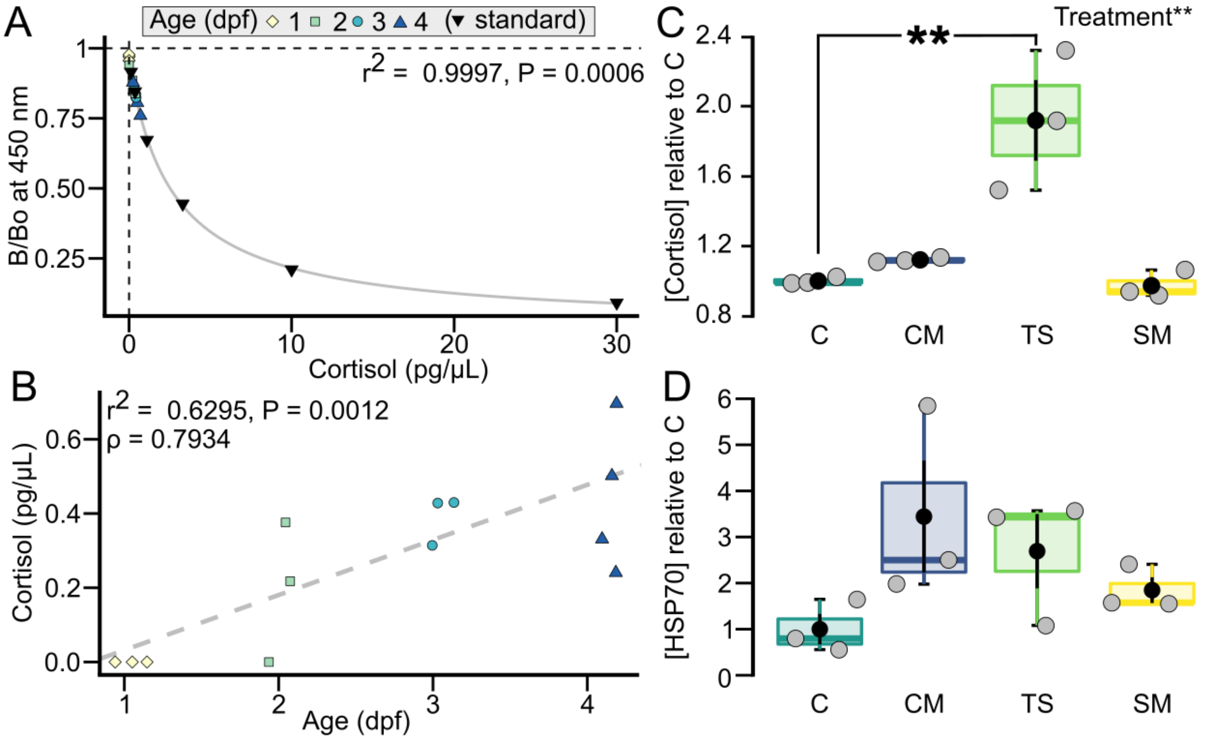

Figure S2. Cortisol levels increase with age and thermal stress in zebrafish embryos but repeated thermal stress inhibits the heat shock protein response at 4 dpf. A) Optimisation of cortisol assay showing in-house extraction method works best with embryos at 4 days post fertilisation (dpf) (dark-blue triangles). Standard curve of cortisol assay (grey line) of the blank-corrected cortisol absorbance (B/Bo, at 450 nm) in function of cortisol standards (pg/µL). Standard curve replicated using the four-parameter nonlinear regression equation computed by mycurvefit.com (fit in top-right corner). Values from control embryos at 1, 2, 3, and 4 dpf (coloured shapes, n = 13, each containing 40 embryos) were interpolated from standards (black diamonds, n = 6). Samples with values outside the detection range (0.07 pg/L, dashed lines) were arbitrarily replaced by "0 pg cortisol/µL". B) Comparison of cortisol levels in zebrafish from 1 to 4 dpf. Fit, significance, and correlation coefficient of linear regression shown in top-left corner by the r-squared, P, and Pearson's rho values respectively. Slope depicted by the grey dashed line. C) Thermal stress induces a cortisol stress response at 4 dpf in zebrafish embryos. Values are expressed as cortisol normalised to protein content and rescaled to control average concentration. Significant effect of treatment (F = 14.35, p = 0.0014) shown on top-right corner. Grey lines show significant comparisons. *: $p \leq 0.05$, **: $p \leq 0.01$. D) Protein expression of heat shock protein 70 (HSP70) normalised to total protein loading (Ponceau staining) and expressed as arbitrary units (a.u.) relative to control C. Open grey circles represent biological replicates values (n = 3 biological replicates containing 60 embryos, total of 180 embryos per treatment). Black circles and thick vertical bars represent mean values ± standard error. C: control in fresh medium at 27°C; CM: control metabolites at 27°C; TS: thermal stress in fresh medium; SM: stress metabolites at 27°C.

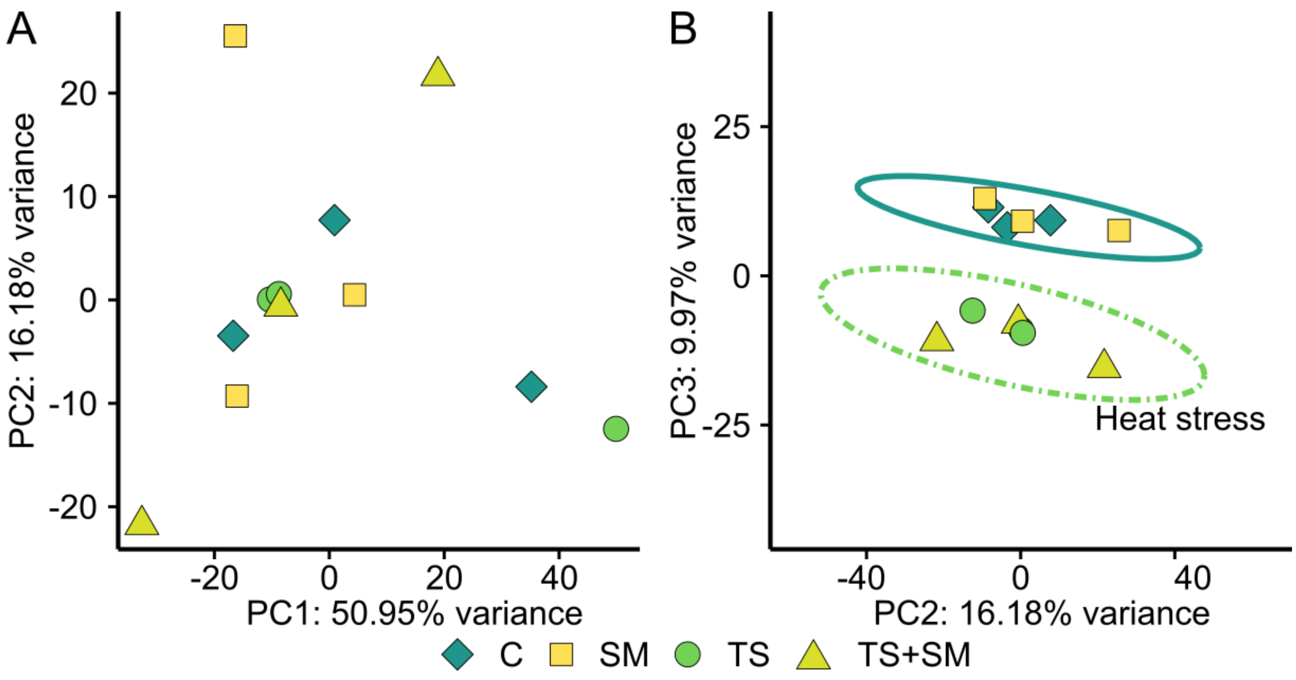

Figure S3. Principal Component Analysis of RNA-seq. A) PCA of axes 1 and 2 and B) 2 and 3. Ellipses show the 95% interval confidence of centroids of heat stress (green dot-dash lines, n = 6 samples) and 27°C (blue solid lines, n = 6 samples). Medium effect is not evident on the first three axes and is not represented. C: control in fresh medium at 27°C, SM: stress metabolites at 27°C, TS: fresh medium in thermal stress, TS+SM: stress metabolites in thermal stress.

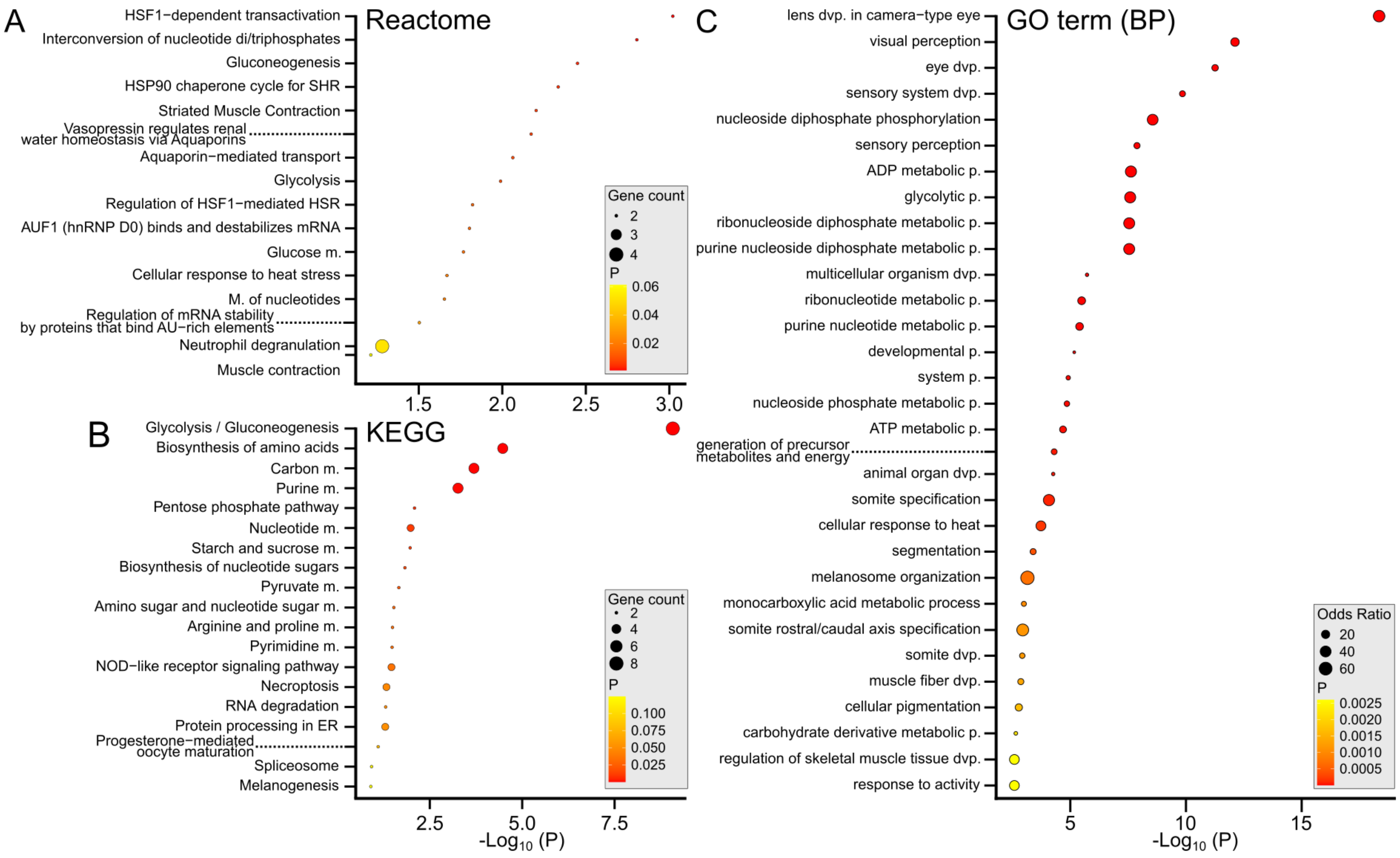

Figure S4. Thermal Stress alters development, metabolism, and stress responses pathways in zebrafish embryos. Functional enrichment of (A) KEGG pathways, (B) Reactome pathways, and (C) Biological Processes (BP) gene ontology (GO) terms of differentially expressed genes in thermal stress (TS) compared to C. All represented terms have ≥ 2 gene counts and are ranked by decreasing significance. Abbreviations: m: metabolism, dvp: development, ER: endoplasmic reticulum, HSR: heat shock response, p: process, SHR: steroid hormone receptor.

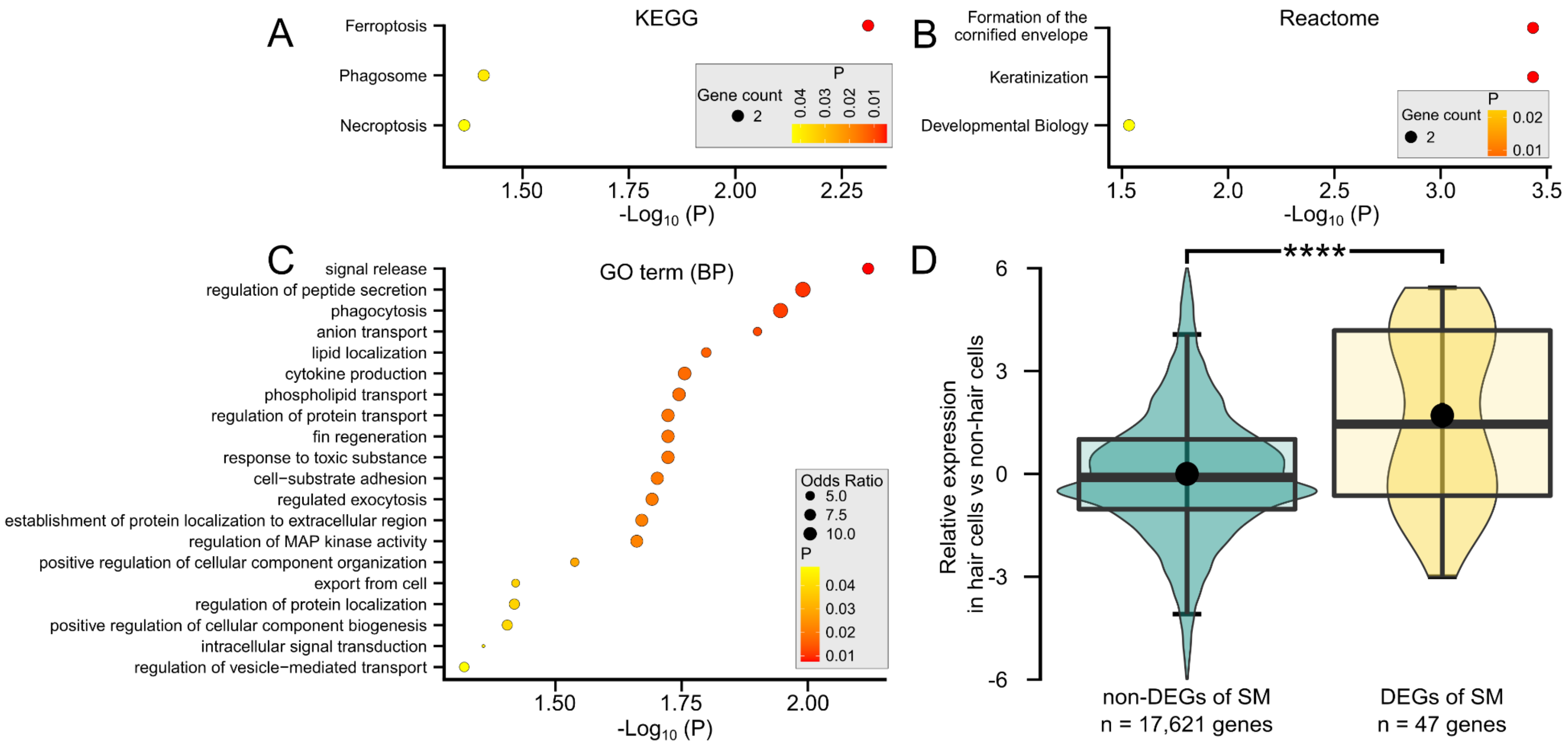

Figure S5. Stress metabolites alter signalling, immunity, and keratan pathways in zebrafish embryos. Functional enrichment of (A) KEGG pathways, (B) Reactome pathways, and (C) Biological Processes (BP) gene ontology (GO) terms of differentially expressed genes in stress metabolites (SM) compared to C. All represented terms have ≥ 2 gene counts and are ranked by decreasing significance. D) Genes of SM are also expressed in sensory hair cells of the lateral line. Relative expression of hair cells compared to non-hair cells of larval zebrafish lateral line were retrieved from Elkon et al. (2015) (28).

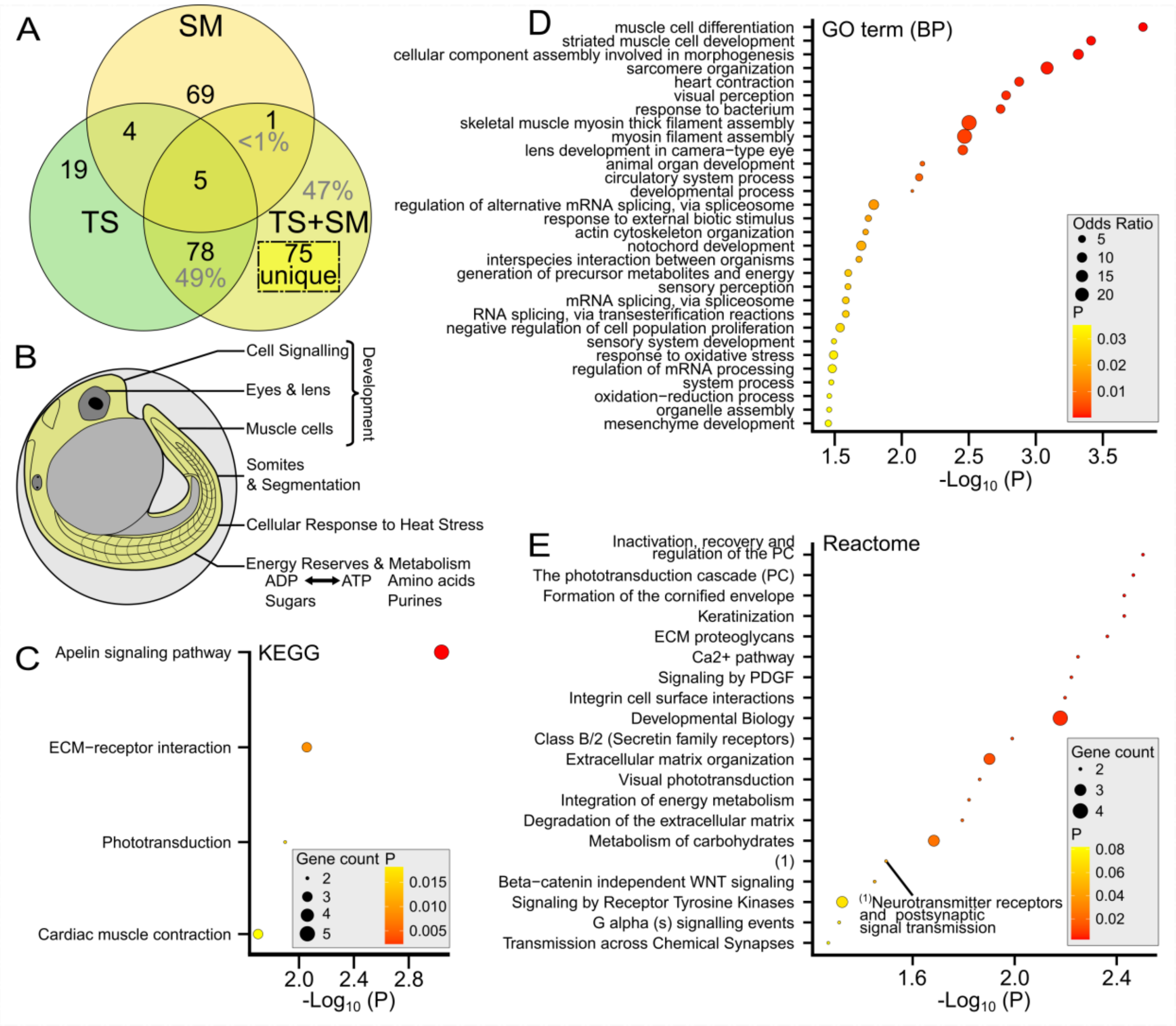

Figure S6. Stress metabolites combined with heat in TS+SM alter unique gene and functions. A) Venn diagram showing the differentially expressed genes shared for each treatment compared to the control C. B) gene functional categories from KEGG, Reactome, and GO Biological Process analysis for all n = 159 DEGs of TS+SM. In C-D-E), genes that are present in TS+SM only (compared to C, n = 75 genes) were retrieved for functional enrichments as they indicate an interactive effect of TS and SM on (C) biological processes gene ontology terms, D) KEGG pathway, and E) Reactome pathways. All represented terms have ≥ 2 gene counts and are ranked by decreasing significance.

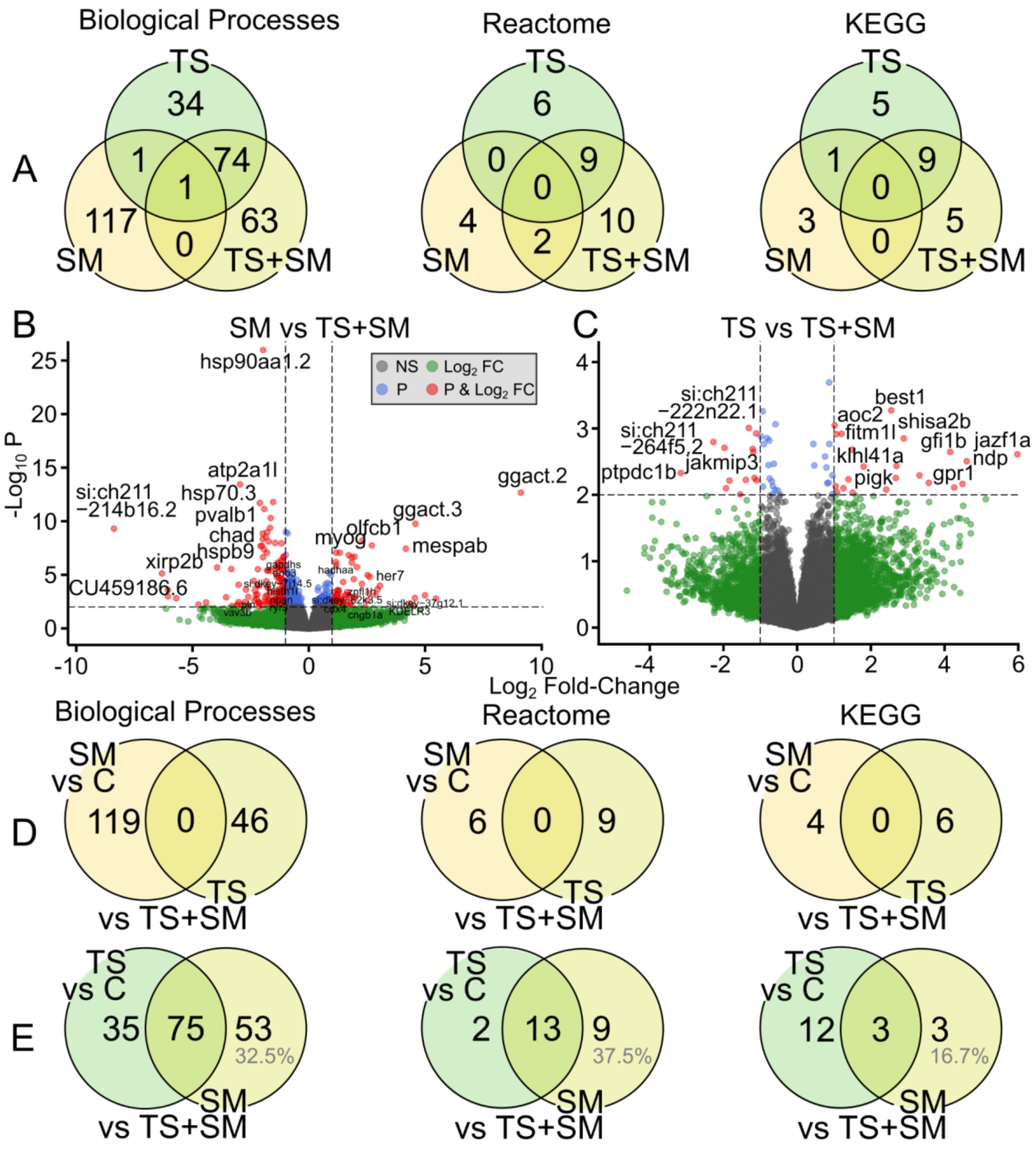

Figure S7. Heat and Stress metabolites induce an interactive effect on TS+SM transcriptome. Row 2: Volcano plot of the differentially expressed genes of (B) SM vs TS+SM and (C) TS vs TS+SM. Genes in red have significant raw p-values (above horizontal line) and an absolute fold change (FC, representing the effect size) greater than 2 ($|\log 2\ FC| > 1$, vertical lines). DEGs left to the left vertical line and right to the right vertical line are respectively significantly underexpressed and overexpressed compared to the control C. Rows 1, 3-4: Venn Diagrams of significant ($p < 0.05$, $n \geq 1$ gene/term) biological process gene ontology terms (left), Reactome (middle), and KEGG pathways (right) that are shared between treatments (A) TS, SM, TS+SM compared to the control C, (D) SM versus C and TS versus TS+SM , and (E) TS versus C and SM versus TS+SM. If there were no interactive effects of TS and SM, one would expect a strong redundancy and shared partitions. In A, B, and C, partitions unique to TS+SM indicate interaction effects in combined treatment TS+SM compared to single treatments TS and SM.

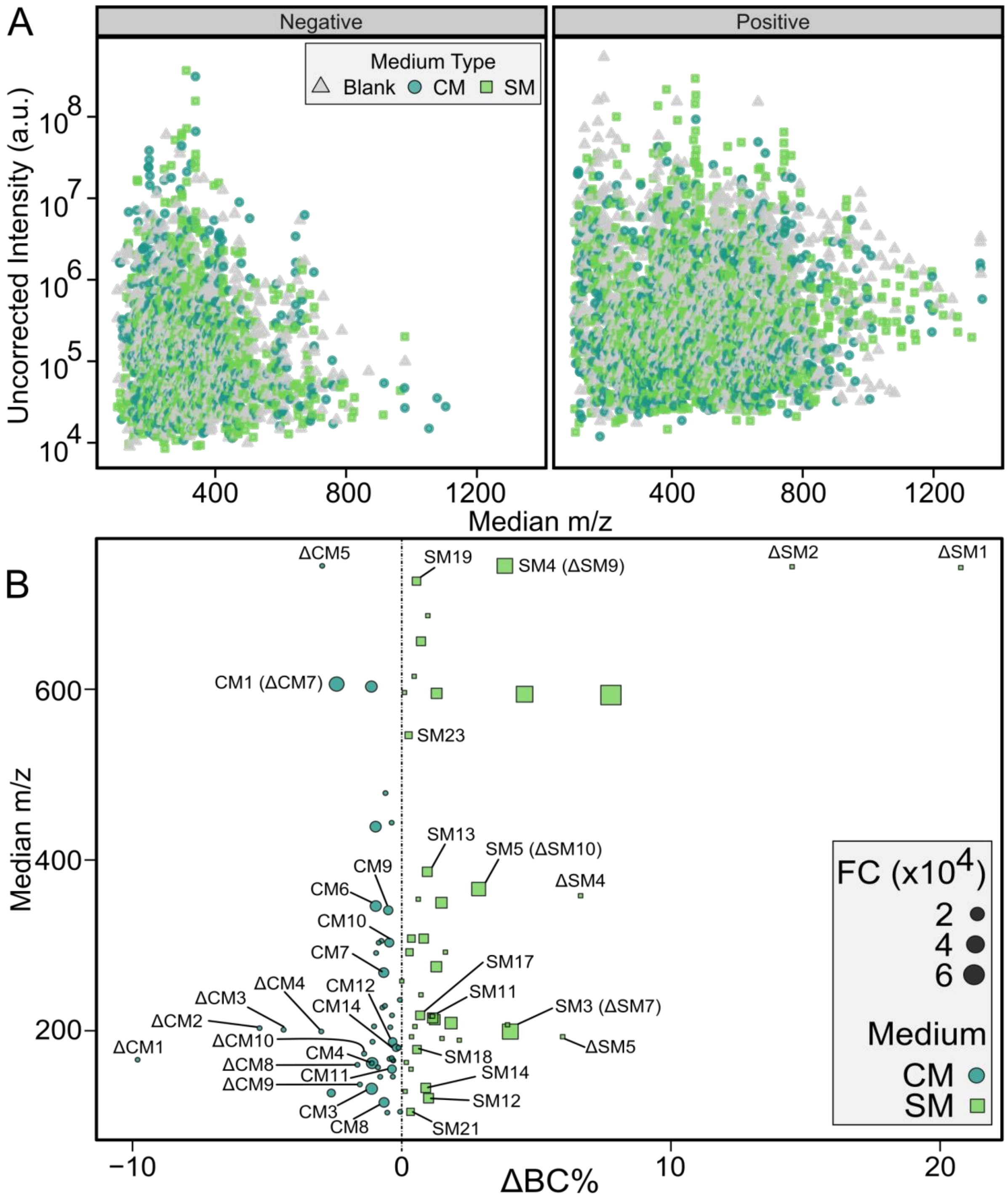

Figure S8. Manhattan and volcano plots of the metabolomic data. A) Raw MS data was preprocessed to yield 5,583 unique masstags in negative (n = 2,238, left) and positive (n = 3,345, right) ion modes. Uncorrected raw intensity areas are displayed in arbitrary units (a.u.). Masstags were assigned to medium types (blank, CM, SM) based on which medium had the highest average intensity. CM: control metabolites, SM: stress metabolites. B) Volcano plot is showing filtered (n = 89) masstags that are possible biomarkers of control metabolites (CM) and stress metabolites (SM) groups (and not present predominantly in the blank) in both negative and positive modes along with their masses (m/z). Data was calculated as blank corrected total percent intensity (BC%) to represent the relative concentrations of the compounds in the sample. Data is presented as the absolute distance of blank corrected total percent intensity between CM and SM. Far-left dots represent CM-specific compounds, and far-right dots represent SM-specific compounds. Labels show the top biomarkers of each group that have possible hits.

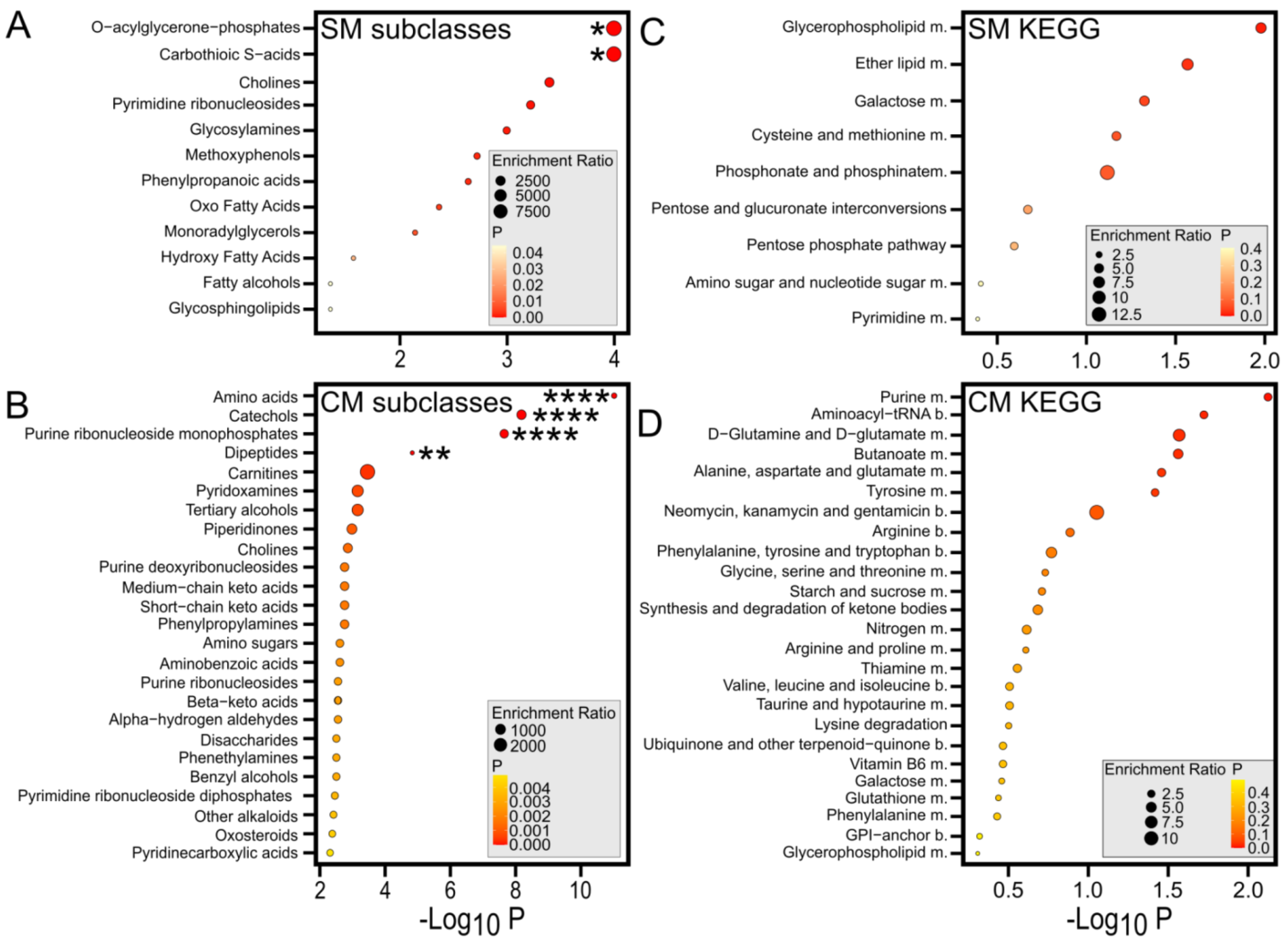

Figure S9. Stress metabolites and control metabolites differ in classes. Subclasses (left) and KEGG pathways (right) functional enrichment for the representative compounds of SM (top row) and CM (bottom row) biomarkers. Only representative compounds assigned to known chemicals were used for the metabolome functional enrichment. Raw p-values are shown. Significant pathways after Holm's p-value correction are shown with *: $p \leq 0.05$, **: $p \leq 0.01$, ****: $p \leq 0.0001$. Abbreviations: b.: biosynthesis, GPI: Glycosylphosphatidylinositol, m.: metabolism.

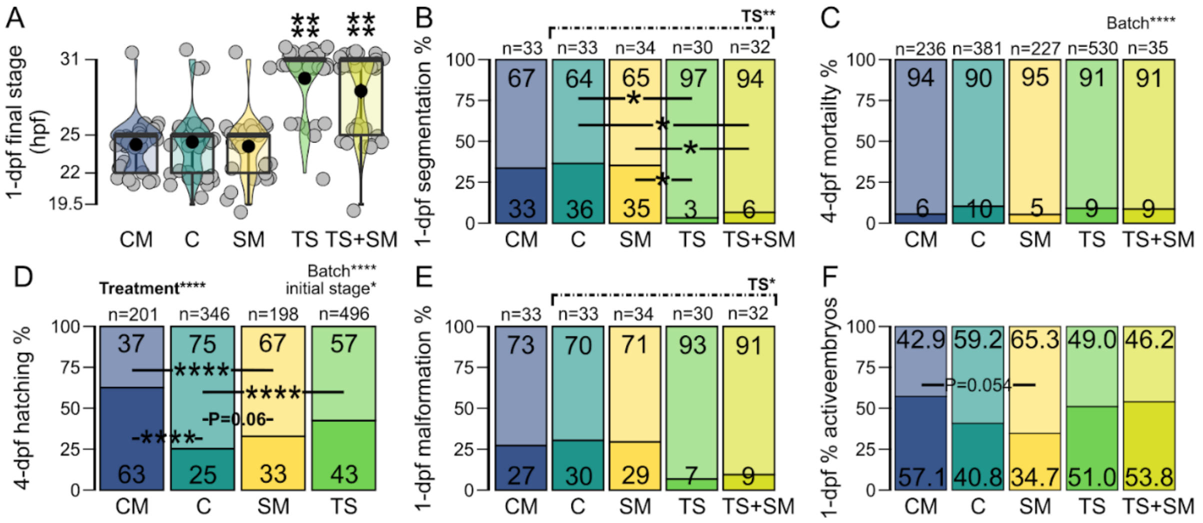

Figure S10. Thermal stress accelerates development at 1 dpf and hatching at 4 dpf. Data was collected at 1 (top row) and 4 (bottom row) days post fertilisation (dpf). CM: control metabolites at 27°C, C: control in fresh medium at 27°C, SM: stress metabolites at 27°C, TS: fresh medium in thermal stress, TS+SM: stress metabolites in thermal stress. A) Final median stage in hours post fertilisation (hpf) at 1 day post fertilisation (dpf). B) Percentage of 1-dpf embryos in segmentation (dark areas) or pharyngula (light areas) periods. C) Percentage of dead (dark areas) or surviving (light areas) at 4 dpf. D) Percentage of hatching (dark areas) or unhatched (light areas) embryos at 4 dpf. E) Percentage of 1-dpf embryos presenting morphological defects (dark areas) or not (light areas). F) Percentage of moving (dark areas) or not (light areas) 1-dpf embryos under acute light change. In B and E, the effects of thermal stress x stress metabolites across C, TS, SM, and TS+SM are shown in top right corners. In C and D, mortality and hatching were recorded over 1,000 embryos across > 30 batches with treatment and covariate effects (batch, initial stage, month) reported above plots when significant. Statistics were computed using Scheirer-Ray-Hare (A) and generalised linear models with binomial distributions (B, C, D). Post-hoc comparisons using Wilcoxon-Mann-Whitney tests (A) and emmeans on models (B, C, D) compared SM, TS, and TS+SM to control C, or SM and C to positive control CM with Tukey correction for false discovery rate. Significant pairwise comparisons are shown by horizontal bars. *: p ≤ 0.05, **: p ≤ 0.01, ***: p ≤ 0.001, ****: p ≤ 0.0001. Samples sizes of top row are shown above treatments in B. Hatching and survival sample sizes are shown above C and D.

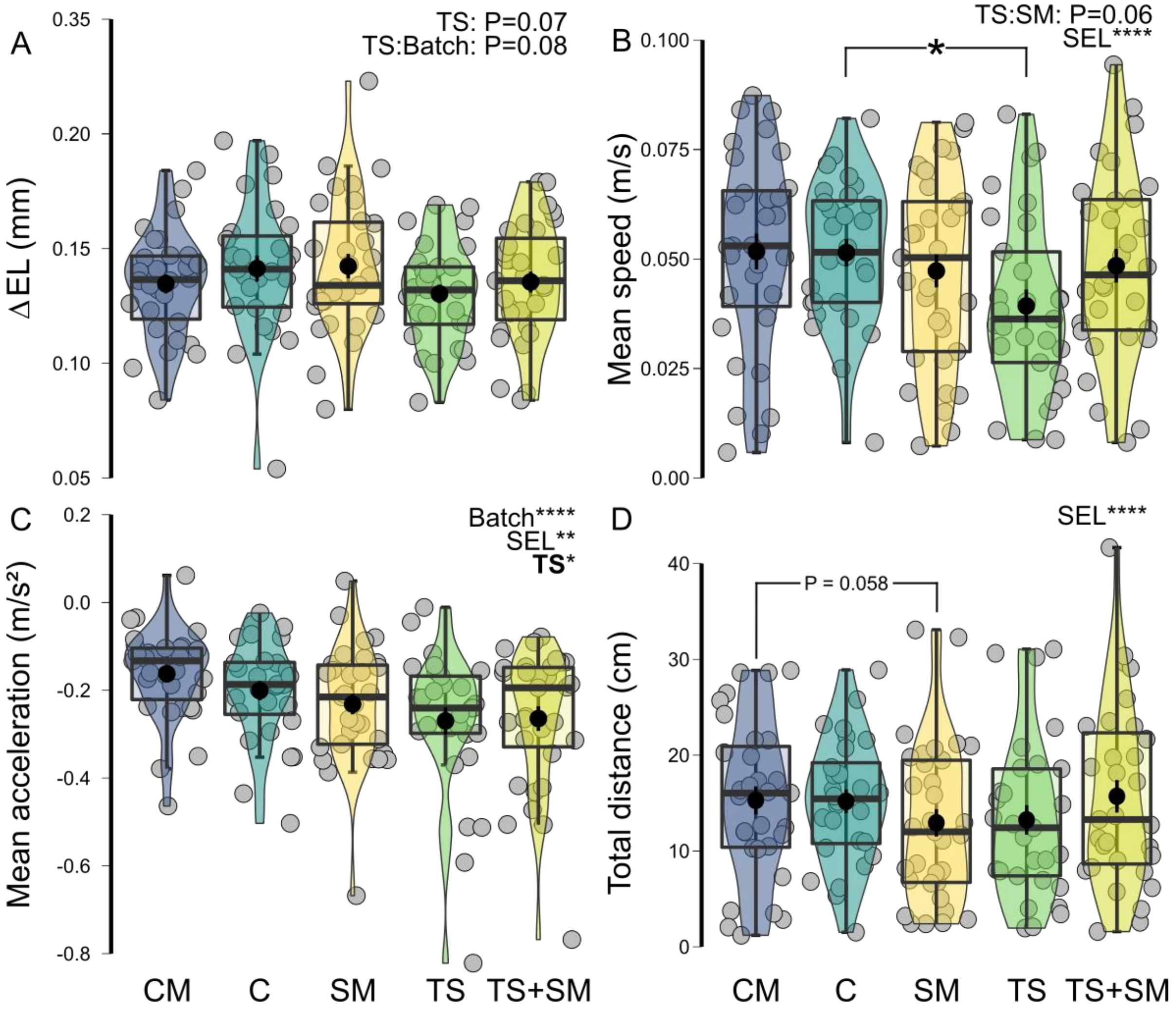

Figure S11. Thermal stress and stress medium alter the phenotypic response in 4-dpf zebrafish. CM: control metabolites at 27°C (n = 31), C: control in fresh medium at 27°C (n = 28), SM: stress metabolites at 27°C (n = 33), TS: fresh medium in thermal stress (n = 29), TS+SM: stress metabolites in thermal stress (n = 31). A) Delta eye length (ΔEL, mm) from 1 to 4 dpf. B) mean speed (m/s) and mean acceleration (C) averaged after the 1st and 3rd stimuli in the touch-evoked swimming behaviour assay at 4 dpf. D) Total distance (cm) after 1st, 2nd, and 3rd stimuli in the touch-evoked swimming behaviour assay at 4 dpf. The effects of thermal stress x stress metabolites across C, TS, SM, and TS+SM are shown in top-right corners, along with covariates (batch, embryo length SEL), when significant. Post-hoc tests compared SM, TS, and TS+SM to control C, or SM and C to positive control CM with Tukey correction for false discovery rate. Significant pairwise comparisons are shown by horizontal bars. *: $p \leq 0.05$, **: $p \leq 0.01$, ***: $p \leq 0.001$, ****: $p \leq 0.0001$.

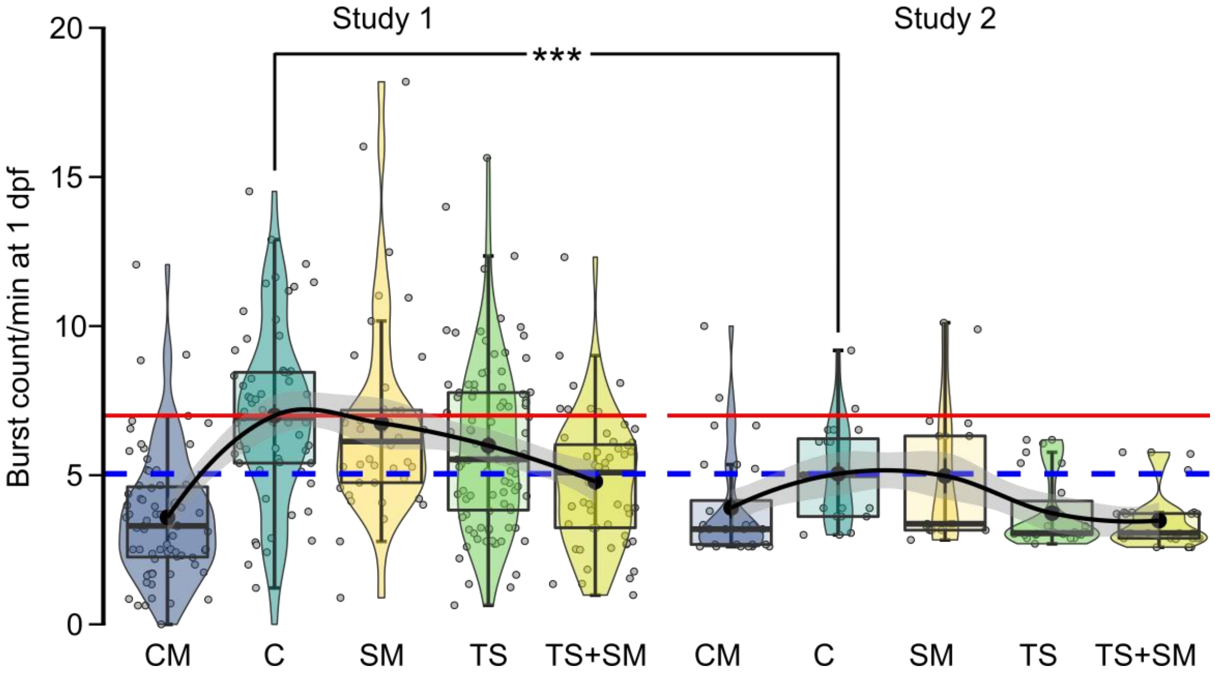

Figure S12. Cross-study comparison of behavioural response to treatments. Study 1: pet-store strain, with 16 hours and 15 min of heat protocol followed by 7 hours and 45 min of recovery period (redrawn from (3). Study 2: AB strain, 24 hours of heat protocol. Inactive embryos were removed in study 2. Both studies tested the acute light-induced startle response at 24 hours. Smoothed lines were added on the arbitrary basis of the gradual developmental effects (in order of increasing final age: CM, C, SM, TS, TS+SM) shown in study 1 and show similar patterns in either study. Asterisks represent the comparison of controls (Welch's modified t-test).

Supplementary Tables

Table S1. Post-hoc pairwise comparisons of cortisol levels. CM: control metabolites at 27°C, C: control in fresh medium at 27°C, SM: stress metabolites at 27°C, TS: fresh medium in thermal stress. Sample size was n = 3 pooled samples of 60 embryos per treatment. Computed using emmeans (7). Effect size interpretation according to Sawilowsky (59). Data collected at 4 days post fertilisation. P-values are corrected for false discovery rate for multiple comparisons. Significant terms are shown in bold.

Contrast	Estimate	SE	df	T ratio	P	Cohen's |d|	Effect size
C-CM	-0.5615	0.379	8	-1.4816	0.4895	6.04	Huge
**C-TS**	-1.8408	0.379	8	-4.8574	**0.0055**	**6.17**	**Huge**
C-SM	0.5219	0.379	8	1.3771	0.5453	0.88	Large
CM-SM	1.0834	0.379	8	2.8587	0.0812	1.85	Very large

Table S2. List of potential stress metabolite candidate chemicals, used for automatic annotation of references from the spectral database. Only candidate chemicals having a registered CID were kept. Candidates were retrieved from literature focusing on stress and animal communication incl. alarm substances and kairomones.

Chemical	Reference	Function	PubChem CID	Structure
Hydrocortisone	(61)	Candidate Stress Metabolite	5754	C21H30O5
Cortisone	(62)	Candidate Stress Metabolite	222786	C21H28O5
Corticotropin	(62)	Candidate Stress Metabolite	16132265	C207H308N56O58S
20β-hydroxycortisone	(62)	Candidate Stress Metabolite	13367714	C21H30O5
L-Arg	(63)	Alarm Substance	6322	C6H14N4O2
L-Leu-L-Leu-OH	(63)	Alarm Substance	76807	C12H24N2O3
Suberic acid	(63)	Alarm Substance	10457	C8H14O4
N-palmitoleyl glutamine	(64)	Kairomone	100937253	C21H38N2O4
N-linoleoyl glutamine	(64)	Kairomone	76970188	C23H40N2O4
N-linolenoyl-glutamine	(64)	Kairomone	5470079	C23H38N2O4
N-olenoyl-glutamine	(64)	Kairomone	52922072	C23H42N2O4
Serotonin	(65)	Candidate Stress Metabolite	5202	C10H12N2O
Glutathione	-	Candidate Stress Metabolite	124886	C10H17N3O6S
Hypoxanthine-3 N-oxide	(66)	Alarm Substance	192963	C5H4N4O2
Chondroitin sulfate	(67)	Alarm Substance	24766	C13H21NO15S
Ichthyopterin	-	Alarm Substance	135449065	C9H11N5O4
7-hydroxybiopterin	(68)	Alarm Substance	50883477	C9H11O4N5
Histamine	-	Candidate Stress Metabolite	774	C5H9N3
Pyridine-N-oxide	(68)	Alarm Substance	12753	C5H5NO
Taurocholic acid	(69)	Social cue	6675	C26H45NO7S
Prostaglandin 2 alpha	(69)	Social cue	448457	C20H32O5
Cadaverine	(69)	Decay cue	273	C5H14N2
L-Cysteine	(69)	Decay cue	5862	C3H7NO2S
Spermine	(69)	Decay cue	1103	C10H26N4
Putrescine	(69)	Alarm Substance	1045	C4H12N2
Acetylcholine	(70)	Alarm Substance	187	C7H16NO2+
Pyrimidine	(71)	Alarm Substance	9260	C4H4N2
Trigonelline	(72, 73)	Candidate Stress Metabolite	5570	C7H7NO2
Homarine	(72, 73)	Candidate Stress Metabolite	3620	C7H7NO2
20-hydroxyecdysone	(74)	Candidate Stress Metabolite	5459840	C27H44O7

Table S3. Possible hits for the top 14 biomarkers of CM cloud and top 23 biomarkers of SM cloud. The order of identifier numbers for CM and SM are based on decreasing CM-to-SM and SM-to-CM fold-change values, respectively. "Unidentified metabolite: found in previous studies but with no annotation. "Unknown masstag": no record in available online databases. Lipid abbreviations indicate the constituent + C-atom double bond. CE: cholesterol ester, DG: diacylglycerol, DGGA: diacylglyceryl glucuronides, FA: fatty acid, MG: monoradyglycerols, PI: phosphatidylinositols, PG: phosphatidylglycerols, PE: phosphatidylethanolamines, PC: phosphatidylcholine, PS: phosphatidylserine, TG: triglycerol, WE: wax monoester. In bold: chemical classification. **A priori* candidate of stress metabolites but found overexpressed in CM. Note: masses may include adducts, see methods for details.

Masstag	ID	Possible hits
**CM compounds**		
M606.0748T37	CM1	**Nucleosides, nucleotides, and analogues:** UDP-N-Acetyl-D-Galactosamine; UDP-N-Acetylglucosamine; UDP-N-Acetyl-D-Mannosamine; **Others:** Unidentified Metabolite
M603.1316T36	CM2	**Others**: Unidentified Metabolite
M132.0769T32	CM3	**Amino acids, peptides, and analogues:** Creatine; Creatinine
M162.1125T28	CM4	**Amino acids, peptides, and analogues:** L-Carnitine; **Lipids***:* FA 7:1;O; WE 7:1;O
M439.0533T39	CM5	Unknown masstag
M346.0558T84	CM6	**Nucleosides, nucleotides, and analogues:** 2'-Deoxyguanosine 3'-Monophosphate; dGMP; 2-Hydroxy-dAMP; 3'-AMP; 7-(5-Phospho-α-D-Ribosyl)Adenine; 9-(2-Deoxy-5-O-Phosphono-β-L-Ribofuranosyl)Guanine; 8-Hydroxy-dAMP; **Monosaccharides:** Adenosine 2'-Phosphate; Adenosine 5'-Monophosphate; **Others:** Unidentified Metabolite
M268.1040T90	CM7	**Nucleosides, nucleotides, and analogues:** Adenosine; Deoxyguanosine
M116.0708T90	CM8	**Amino acids, peptides, and analogues:** L-Proline; D-Proline; **Lipid**: FA 5:2
M341.1089T38	CM9	**Lipids and lipid-like molecules (fatty acyl glycosides):** 3-B-Galactopyranosyl Glucose; α-D-Glucosyl-(1->4)-Aldehydo-D-Mannose; β-D-Glucosyl-(1->4)-Aldehydo-D-Mannose; D-Glucopyranosyl-(1->4)-Aldehydo-D-Mannose; Kojibiose; Turanose; Maltulose; **Carbohydrates and carbohydrate conjugates:** Sucrose; Lactose; Galactinol; Isomaltose; Epimelibiose; 6-O-α-D-Glucopyranosyl-α-D-Fructofuranose; 6-O-α-D-Glucopyranosyl-β-D-Fructofuranose; α,β-Trehalose; α-D-Aldosyl β-D-Fructoside; Galabiose; α-D-Galactosyl-(1->2)-α-D-Galactose; α-D-Galactosyl-(1->2)-β-D-Galactose; α-D-Galactosyl-(1->2)-D-Galactose; α-D-Galactosyl-(1->3)-α-D-Galactose; α-D-Galactosyl-(1->3)-β-D-Mannose; α-D-Galactosyl-(1->3)-D-Galactose; α-D-Galp-(1->3)-β-D-Galp; α-D-Galp-(1->4)-β-D-Galp; α-D-Galp-(1->6)-D-Galp; α-D-Glcp-(1->2)-β-D-Galp; α-D-Glcp-(1->3)-β-D-Glcp; α-D-Glcp-(1->6)-D-Manp; α-D-Glucosyl-(1->2)-D-Mannose; α-D-Glucosyl-(1,3)-D-Mannose; α-D-Glucosyl-(1->3)-D-Mannopyranose; α-D-Glucosyl-(1->4)-β-D-Mannose; α-D-Glucosyl-(1->4)-D-Mannopyranose; α-D-Manp-(1->2)-α-D-Manp; α-D-Manp-(1->2)-D-Manp;3-O-α-D-Mannopyranosyl-α-D-Mannopyranose; Mannobiose; α-D-Manp-(1->6)-β-D-Manp; Neoglyco Interleukin-1a; β-(1->3)-Galactobiose; β-(1->6)-Galactobiose; β,β-Trehalose;Mannosylfructose; β-D-Gal-(1->4)-D-Man;β-D-Galactopyranosyl-(1->4)-α-D-Galactopyranose; β-D-Galactopyranosyl-(1->4)-D-Galactopyranose; β-D-Galactopyranosyl-(1->6)-β-D-Mannopyranose; β-D-Galactosyl-(1->3)-α-D-Mannose;β-D-Galf-(1->2)-D-Manp; β-D-Galf-(1->3)-α-D-Manp; β-D-Galp-(1->2)-β-D-Galp; β-D-Galp-(1->6)-D-Galp; β-D-Glcp-(1->2)-α-D-Manp; β-D-Glcp-(1->2)-β-D-Galp; β-D-Glucosyl-(1->4)-α-D-Mannose; β-D-Glucosyl-(1->4)-β-D-Mannose; β-D-Glucosyl-(1->4)-D-Mannopyranose; β-D-Manp-(1->2)-α-D-Manp; β-1,2-Mannobiose; D-Fructosyl-D-Fructofuranose; D-Galactosyl-(1->4)-β-D-Glucose; D-Glucopyranosyl-(1->3)-D-Mannopyranose; D-Glucopyranosyl-(1->4)-D-Mannopyranose; Laminarabiose; Nigerose; Palatinose; Trehalose; α-Maltose; β-Cellobiose; β-Maltose; Maltose; Melibiose; Sophorose; Neotrehalose; β-Lactose; Arabinofuranobiose; Galactose; **Others**: C12H22O11-Disaccharide-(6C/6C; Glc-Glc/Glc-Frc/Gal-Glc); Sucrose And Disaccharides-13C0[-H+]-; Unidentified Metabolite

Table S3. Continued.

Masstag	ID	Possible hits
M303.2175T212	CM10	**Lipids and lipid-like molecules:** Ustilic Acid B; 11,12,15-Trihydroxy Palmitic Acid; 2,15,16-Trihydroxy Palmitic Acid; 8,9,16-Trihydroxy Palmitic Acid; Aleuritic Acid; 2-Hydroxymyristic Acid; 9-Hydroxymyristic Acid; 10-Hydroxytetradecanoic Acid; 11-Hydroxytetradecanoic Acid; 11S-Hydroxytetradecanoic Acid; 3-Hydroxytetradecanoic Acid; 5-Hydroxytetradecanoic Acid; 6-Hydroxytetradecanoic Acid; 6R-Hydroxytetradecanoic Acid; 2S-Hydroxytetradecanoic Acid; Omega-Hydroxy Myristic Acid; (R)-3-Hydroxytetradecanoic Acid; 2-Methoxytetradecanoic Acid; 3R-Hydroxymyristic Acid Methyl Ester; 9-Hydroxy-Pentadecanoic Acid; 15-Hydroxypentadecanoic Acid; 2-Hydroxypentadecanoic Acid; 2R-Hydroxypentadecanoic Acid; 3-Hydroxypentadecanoic Acid; 3R-Hydroxypentadecanoic Acid; 3S-Hydroxypentadecanoic Acid; 4-Hydroxypentadecanoic Acid; Ethyl 3-Hydroxydodecanoate; FA 16:0;O3; MG 13:0;O; FA 15:0;O; MG O-12:1; FA 14:0;O; MG O-11:1; **Others:** Unidentified Metabolite;
M155.0816T28	CM11	**Alkaloids and derivatives:** Trigonelline*; **Amino acids, peptides, and analogues:** Glycyl-Tyrosine; Tyrosyl-Glycine; **Benzenoids**: Anthranilate; **Pyridines and derivatives:** Homarine*
M187.1077T30	CM12	**Amino acids, peptides, and analogues:** Pro-Ala; N(5)-(L-1-Carboxyethyl)-L-Ornithine; N(6)-Carboxymethyl-L-Lysine;Ser-Val; Val-Ser; (Ac)2-L-Lysyl-D-Alanyl-D-Alanine; Glu-Pro-Lys; Pro-Glu-Lys; 2-Amino-5-Oxohexanoic Acid; 4-Acetamidobutanoic Acid; Isobutyrylglycine; L-Allysine; 5-Hydroxypipecolic Acid; N-Butyrylglycine; Gly-Pro; Pro-Gly; Prolyl-Alanine; Alanyl-Proline; **Keto acids and derivatives**: 2-Keto-6-Aminocaproate; (5S)-5-Amino-3-Oxohexanoic Acid; Glu-Lys-Pro; Lys-Glu-Pro; Lys-Pro-Glu; Pro-Lys-Glu; (4R)-5-Oxo-L-Leucine;<br>**Carbonyl compounds**: 1-Ethyl-1H-Pyrrole-2-Carboxaldehyde; **Benzenediols**: 5-Hydroxydopamine; Norepinephrine;<br>**Pyridines and derivatives**: 6-Acetyl-2,3-Dihydro-2-(Hydroxymethyl)-4(1H)-Pyridinone; Ala-Pro; Pyridoxine
M127.0728T33	CM13	Unknown masstag
M180.0667T83	CM14	**Amino acids, peptides, and analogues:** L-Tyrosine
**SM compounds**		
M593.1612T383	SM1	Unknown masstag
M594.1646T383	SM2	**Others**: Unidentified Metabolite
M199.0047T124	SM3	**Lipids and lipid-like molecules:** Dihydroxyacetone Phosphate Acyl Ester; **Others**: Unidentified Metabolite
M744.5022T467	SM4	**Lipids and lipid-like molecules:** DGGA 31:1;O; PI(O-14:0/13:0); PI(O-16:0/11:0); PG(25:0/4:0); PG(27:0/2:0); PG(12:0/17:0); PG(13:0/16:0); PG(14:0/15:0); PG(15:0/14:0); PG(16:0/13:0); PG(17:0/12:0); **Others:** Unidentified Metabolite; **UniProt proteins:** Spondin-2 A0A1Y8Ely7 of *Homo sapiens* (A0A1Y8Ely7_Human); Bbrv_Locus22923 Of *Bracon brevicornis* A0A6V7Ii27 (A0A6V7Ii27_9Hyme); Bzw1 Of Prickly gecko *Heteronotia binoei* A0A0U3Jgs3 (A0A0U3Jgs3_9Saur).
M365.9712T82	SM5	**Lipids:** TG 69:8;O and TG O-69:9;O2
M208.9947T164	SM6	Unidentified Metabolite
M349.9763T124	SM7	Unknown masstag
M595.1675T383	SM8	**Others**: Unidentified Metabolite
M274.9905T124	SM9	**Others**: Unidentified Metabolite
M213.0121T189	SM10	**Others**: Unidentified Metabolite
M214.9994T82	SM11	**Lipids and lipid-like molecules:** 2-Oxo-4-Methylthiobutanoic Acid; **Other:** Unidentified Metabolite

Table S3. Continued.

Masstag	ID	Possible hits
M120.9964T82	SM12	**Organic acids and derivatives:** Thioacetate; **Organic oxygen compounds**: 3-Mercaptolactate
M386.1569T153	SM13	**Tripeptides**: Met-Pro-Thr; Met-Thr-Pro; Pro-Met-Thr; Pro-Thr-Met; Thr-Met-Pro; Thr-Pro-Met; Asp-Pro-Pro; Pro-Asp-Pro; Pro-Pro-Asp; Glu-Pro-Pro; Pro-Glu-Pro; Pro-Pro-Glu
M133.0507T72	SM14	**Lipids and lipid-like molecules**: 2,3-Dihydroxy-2-Methylbutanoic Acid;(R)-2,3-Dihydroxy-Isovalerate;2,3-Dihydroxyisovaleric Acid;2,3-Dihydroxy-Valeric Acid; (R)-Glycerol 1-Acetate; **Organic oxygen compounds:** 1-Deoxy-D-Xylulose;Deoxyribose;5-Deoxy-D-Ribose;Xylitol; Deoxyribose; **Organoheterocyclic compounds:** 2-Deoxy-α-D-Ribopyranose;2-Deoxy-L-Arabinose; **Lipids:** FA 5:0;O2;FA 4:0;WE 4:0;FA 3:0
M307.9657T124	SM15	Unknown masstag
M656.1570T383	SM16	**Others**: Unidentified Metabolite
M218.0356T27	SM17	**Organic nitrogen compounds:** Phosphocholine; **Other**: Unidentified Metabolite
M178.0262T124	SM18	**Organic oxygen compounds:** α-D-Kdo-4P-Oall; α-D-Kdo-5P-Oall; **Other**: Unidentified Metabolite
M726.6238T461	SM19	**Lipids and lipid-like molecules:** GalCer(D14:0/23:0); GalCer(D17:0/20:0); GlcCer(D14:0/23:0); GlcCer(D17:0/20:0); DG(17:0/22:2(13Z,16Z)/0:0); DG(17:1(9Z)/22:1(13Z)/0:0); DG(17:2(9Z,12Z)/22:0/0:0); DG(18:2(9Z,12Z)/21:0/0:0); DG(19:0/20:2(11Z,14Z)/0:0); DG(19:1(9Z)/20:1(11Z)/0:0); DGTS(16:0/16:0); GalCer(D19:0/18:0);GlcCer(D19:0/18:0); N-Glycoloylganglioside Gm2; Cholest-5-En-3Beta-Yl (7Z,10Z,13Z,16Z,19Z-Docosapentaenoate); CE(22:5(4Z,7Z,10Z,13Z,16Z)); CE(22:5(7Z,10Z,13Z,16Z,19Z)); CE(22:3); GalCer(D15:0/22:0); GalCer(D16:0/21:0); GalCer(D18:0/19:0); GalCer(D20:0/17:0); GalCer(D21:0/16:0); GalCer(D22:0/15:0); GlcCer(D15:0/22:0); GlcCer(D16:0/21:0); GlcCer(D18:0/19:0); GlcCer(D20:0/17:0); GlcCer(D21:0/16:0); GlcCer(D22:0/15:0); **UniProt Protein**: Uncharacterized Protein From Gobi Fish, Protein Contains Lim Domain, Blast Hit Zebrafish Gene Ldb1A
M307.9647T82	SM20	**Others**: Unidentified Metabolite
M105.0007T82	SM21	**Organic oxygen compounds:** 3-Mercaptolactate
M291.9702T124	SM22	Unknown masstag
M546.1609T495	SM23	**UniProt Protein**: Fungal Cutinase 2; Scorpion Toxin Tetrapandin-2; **Others**: Unidentified Metabolite

Table S4. Permutational multivariate analysis of variance of morphological data at 1 day post fertilisation. Two-way PERMANOVA representing the effects of thermal stress (TS) and stress metabolites (SM). Effect size interpretation according to Sawilowsky (59). Only complete cases for all the response variables are analysed. N = 999 permutations. Response variables: otic vesicle length (OVL), eye length (EL), shortest embryo length (SEL), longest embryo length (LEL), dorsoventral length (DVL), yolk extension length (YEL), yolk ball length (YBL), tail width (TW), yolk extension-to-yolk ball ratio (YE/YB), head-trunk angle (HTA), eye area (EA), whole-body area (WBA), yolk extension area (YEA), yolk ball area (YBA), Final stage (hours post fertilisation), defects %, final stage period (segmentation or pharyngula). Adjusted p-values are given for post-hoc comparisons. Significant terms are shown with p-values in bold.

Term	Df	Sum sq.	Mean sq.	F	R2	P
2-way PERMANOVA						
**TS**	1	2432.2669	2432.2669	**30.1022**	**0.2475**	**0.0010**
SM	1	13.0015	13.0015	0.1609	0.0013	0.7610
TS:SM	1	189.0455	189.0455	2.3397	0.0192	0.1320
Residuals	89	7191.2206	80.80002	-	0.7319	-
Total	92	9825.5346	-	-	1.0000	-
Post-hoc comparisons						
**TS-C**	1	0.0395	-	**23.2814**	**0.3360**	**0.0015**
SM-C	1	0.0019	-	1.1101	0.0316	0.3050
**(TS+SM)-C**	1	0.0295	-	**17.9542**	**0.2852**	**0.0015**
CM-C	1	-4E-4	-	-0.2211	-0.0059	0.9960
CM-SM	1	-5E-4	-	0.3231	0.0089	0.9960

Table S5. Analysis of variance of increment in embryo size (ΔSEL) between 1 and 4 days post fertilisation. Two-way ANOVA representing the effects of thermal stress (TS) and stress metabolites (SM). Effect size interpretation according to Sawilowsky (59). P-values are corrected for false discovery rate for multiple comparisons. Significant terms are shown with p-values in bold.

Term	df	Sum Sq.	Mean Sq.	Statistic	P	Cohen's |d|	Effect size
**TS**	1	1.2520	1.2520	20.9948	**< 0.0001**	**0.81**	**Large**
**SM**	1	0.3969	0.3969	6.6546	**0.0112**	**0.43**	**Small**
**Batch**	1	0.3997	0.3997	6.7027	**0.0109**	**0.44**	**Small**
TS:SM	1	0.0021	0.0021	0.0355	0.8509	0.03	Tiny
Residuals	114	6.7985	0.0596	NA	NA		

Table S6. Post-hoc comparison of increment in embryo size (ΔSEL) between 1 and 4 days post fertilisation. CM: control metabolites at 27°C (n = 31), C: control in fresh medium at 27°C (n = 28), SM: stress metabolites at 27°C (n = 33), TS: fresh medium in thermal stress (n = 29), TS+SM: stress metabolites in thermal stress (n = 31). Effect size interpretation according to Sawilowsky (59). Significant terms are shown with p-values in bold.

Contrast	Estimate	SE	df	T ratio	P	Cohen's |d|	Effect size
SM - C	0.1037	0.0637	114	1.6280	0.1369	0.38	Small
**TS - C**	-0.2160	0.0647	114	-3.3381	**0.0034**	**0.78**	**Medium**
(TS+SM) - C	-0.0954	0.0637	114	-1.4980	0.1369	0.34	Small
C - CM	-0.0061	0.0697	85	-0.0873	0.9307	0.02	Tiny
SM - CM	0.0971	0.0679	85	1.4291	0.3133	0.40	Small

Table S7. Analysis of variance of burst count per minute at 1 day post fertilisation. Two-way ANOVA representing the effects of thermal stress (TS) and stress metabolites (SM). Effect size interpretation according to Sawilowsky (59). Only active embryos (burst count per min > 0) are analysed. Significant terms are shown with p-values in bold.

Term	df	Sum sq.	Mean sq.	Statistic	P	Cohen's |d|	Effect size
**TS**	1	44.0656	44.0656	**18.1540**	**< 0.0001**	**0.91**	**Large**
SM	1	0.7134	0.7134	0.2939	0.5891	0.11	Very small
TS:SM	1	0.1780	0.1780	0.0733	0.7872	0.05	Tiny
Residuals	87	211.1766	2.4273	-	-	-	-

Table S8. Post-hoc comparison of burst count per minute at 1 day post fertilisation. CM: control metabolites at 27°C (n = 28), C: control in fresh medium at 27°C (n = 20), SM: stress metabolites at 27°C (n = 17), TS: fresh medium in thermal stress (n = 26), TS+SM: stress metabolites in thermal stress (n = 28). P-values are corrected by false discovery rate for multiple comparisons. Only active embryos (burst count per min > 0) are analysed. Effect size interpretation according to Sawilowsky (59). Significant terms are shown with p-values in bold.

Contrast	Estimate	SE	df	T ratio	P	Cohen's |d|	Effect size
SM-C	-0.0701	0.5140	87	-0.1363	0.8919	0.03	Tiny
**TS-C**	-1.3190	0.4634	87	**-2.8464**	**0.0083**	**0.91**	**Large**
**(TS+SM)-C**	-1.5695	0.4561	87	**-3.4409**	**0.0027**	**1.19**	**Large**
C-CM	1.141	0.577	62	1.9776	0.0821	0.64	Medium
SM-CM	1.071	0.606	62	1.7674	0.0821	0.51	Medium

Table S9. Stress Metabolites alter the expression of *chs1* and *prg4a* in a social environment. C: control in fresh medium at 27°C, SM: stress metabolites at 27°C. Sample size was n = 4 pooled samples of 20 embryos per treatment. Computed using Student's t-tests with effect size interpretation according to Sawilowsky (59). Data collected at 1 day post fertilisation using Loop-Mediated Isothermal Amplification (LAMP). Significant genes are shown in bold. c*hs1*: chitin synthase 1, *ldha*: lactate dehydrogenase A4, *ora3*: olfactory receptor class A related 3* (undetected in SM), *otofa*: otoferlin a, *prg4a*: proteoglycan 4a, *tlr18*: toll-like receptor 18.

Contrast	Gene	T ratio	P	Cohen's |d|	Effect size
**C-SM**	***chs1***	3.08	**0.0487**	**2.18**	**Huge**
C-SM	*ldha*	-0.14	0.8960	-0.10	Tiny
C-SM	*ora3**	-	-	-	-
C-SM	*otofa*	-1.67	0.1760	-1.18	Large
**C-SM**	***prg4a***	-8.01	**0.0026**	**-6.52**	**Huge**
C-SM	*tlr18*	-1.32	0.2410	-0.93	Large

Table S10. Summary statistics of read trimming and filtering using FASTP.

ID	reads passed filter	reads failed due to low quality	reads failed due to too many N	reads failed due to too short	reads with adapter trimmed	bases trimmed due to adapters	reads with polyX in 3' end	bases trimmed in polyX tail	Duplication Rate
C1	66789368	2049424	6380	778802	46694	465998	742026	12735945	33.24%
C2	70157084	1075008	6612	780074	48594	481142	426379	5924608	32.92%
C3	60700490	966016	5630	638096	50684	478593	348080	5036478	24.04%
SM1	48128746	875084	4592	660968	28160	280942	353054	5606489	31.16%
SM2	54513078	1355330	5014	647444	35470	363886	442841	6961920	26.90%
SM3	65615614	2395322	6218	1519434	52446	510141	765278	15618099	35.27%
TS1	75209810	2199102	7322	1370030	55204	532218	747925	13281926	35.07%
TS2	53527434	1381108	5216	741882	31900	329816	434426	6785843	31.75%
TS3	78091254	1463140	7226	755040	83962	724661	480490	7501295	24.53%
TS+SM1	1.47E+08	1980930	10298	10536926	148896	1299780	1088457	17690732	60.52%
TS+SM2	56127814	1942186	4958	1938192	45272	422309	510880	8524848	31.97%
TS+SM3	52104876	1172360	4786	505202	44110	434657	413575	6777427	25.80%

Table S11. Summary statistics of STAR alignment retrieved from STAR log files.
"reads unmapped: too many mismatches" and "chimeric reads" were 0% for all samples. "% of reads unmapped: too many mismatches" and "% of chimeric reads" were 0% for all samples.

ID	N reads	N uniquely mapped	N total splices	N multimapped	N mapped to too many loci	% of reads unmapped: too short	% of reads unmapped: other
C1	33394684	30476105 (91.26%)	8458103	1592839 (4.77%)	171886 (0.51%)	3.30%	0.15%
C2	35078542	30004837 (85.54%)	8497405	3641252 (10.38%	345026 (0.98%)	2.95%	0.15%
C3	30350245	27573235 (90.85%)	9327410	1663236 (5.48%)	132617 (0.44%)	3.11%	0.13%
SM1	24064373	21485075 (89.28%)	6604538	1664884 (6.92%)	143939 (0.6%	3.07%	0.13%
SM2	27256539	24388154 (89.48%)	7297002	1671412 (6.13%)	148822 (0.55%)	3.71%	0.14%
SM3	32807807	29009287 (88.42%)	7800715	2335967 (7.12%)	238104 (0.73%)	3.55%	0.18%
TS1	37604905	34075018 (90.61%)	10074769	2020917 (5.37%)	171724 (0.46%)	3.42%	0.14%
TS2	26763717	24363680 (91.03%)	7309245	1387310 (5.18%)	121582 (0.45%)	3.20%	0.13%
TS3	39045627	35653282 (91.31%)	13001237	2068406 (5.3%)	135920 (0.35%)	2.91%	0.13%
TS+SM1	73740522	68107640 (92.36%)	21136475	3322856 (4.51%)	280297 (0.38%)	2.64%	0.11%
TS+SM2	28063907	25266454 (90.03%)	7597755	1708707 (6.09%)	141028 (0.5%)	3.25%	0.13%
TS+SM3	26052438	22690806 (87.1%)	6594073	2157849	177209	3.76%	0.18%

Table S12. Samtools alignment summary statistics of "aligned to transcriptome" alignment file.

ID	Properly paired	With itself and mate mapped	singletons
C1	50428382 + 0 (100%: N/A)	50428382 + 0	0 + 0 (0.00%)
C2	50027650 + 0 (100%: N/A)	50027650 + 0	0 + 0 (0.00%)
C3	46691920 + 0 (100%: N/A)	46691920 + 0	0 + 0 (0.00%)
SM1	36381838 + 0 (100%: N/A)	36381838 + 0	0 + 0 (0.00%)
SM2	40816314 + 0 (100%: N/A)	40816314 + 0	0 + 0 (0.00%)
SM3	48357664 + 0 (100%: N/A)	48357664 + 0	0 + 0 (0.00%)
TS1	57071652 + 0 (100%: N/A)	57071652 + 0	0 + 0 (0.00%)
TS2	40974260 + 0 (100%: N/A)	40974260 + 0	0 + 0 (0.00%)
TS3	61461660 + 0 (100%: N/A)	61461660 + 0	0 + 0 (0.00%)
TS+SM1	116291962 + 0 (100%: N/A)	116291962 + 0	0 + 0 (0.00%)
TS+SM2	42623210 + 0 (100%: N/A)	42623210 + 0	0 + 0 (0.00%)
TS+SM3	37492778 + 0 (100%: N/A)	37492778 + 0	0 + 0 (0.00%)

Supplementary References

1. N. Villamizar, L. M. Vera, N. S. Foulkes, F. J. Sánchez-Vázquez, Effect of lighting conditions on zebrafish growth and development. Zebrafish 11, 173–181 (2014).
2. G. R. Scott, I. A. Johnston, Temperature during embryonic development has persistent effects on thermal acclimation capacity in zebrafish. Proc. Natl. Acad. Sci. U. S. A. 109, 14247–14252 (2012).
3. L. Feugere, V. F. Scott, Q. Rodriguez-Barucg, P. Beltran-Alvarez, K. C. Wollenberg Valero, Thermal stress induces a positive phenotypic and molecular feedback loop in zebrafish embryos. J. Therm. Biol. 102, 103114 (2021).
4. Cold Spring Harbor Laboratory Press, E3 medium (for zebrafish embryos). Cold Spring Harb. Protoc. 2011, db.rec66449 (2011).
5. K. S. Wilson, et al., Physiological roles of glucocorticoids during early embryonic development of the zebrafish (*Danio rerio*). J. Physiol. 591, 6209–6220 (2013).
6. R. A. Peterson, J. E. Cavanaugh, Ordered quantile normalization: a semiparametric transformation built for the cross-validation era. J. Appl. Stat. 47, 2312–2327 (2020).
7. R. V. Lenth, emmeans: Estimated Marginal Means, aka Least-Squares Means (2022).
8. R Core Team, R: A Language and Environment for Statistical Computing (2020).
9. S. E. Walker, J. Lorsch, "Chapter Nineteen - RNA Purification – Precipitation Methods" in Methods in Enzymology, J. Lorsch, Ed. (Academic Press, 2013), pp. 337–343.
10. S. Chen, Y. Zhou, Y. Chen, J. Gu, fastp: an ultra-fast all-in-one FASTQ preprocessor. Bioinformatics 34, i884–i890 (2018).
11. A. Dobin, et al., STAR: ultrafast universal RNA-seq aligner. Bioinformatics 29, 15–21 (2013).
12. S. Sayols, D. Scherzinger, H. Klein, dupRadar: a Bioconductor package for the assessment of PCR artifacts in RNA-Seq data. BMC Bioinformatics 17, 428 (2016).
13. M. I. Love, W. Huber, S. Anders, Moderated estimation of fold change and dispersion for RNA-seq data with DESeq2. Genome Biol. 15, 550 (2014).
14. M. Morgan, BiocManager: Access the Bioconductor Project Package Repository (2021).
15. M. Morgan, V. Obenchain, J. Hester, H. Pagès, SummarizedExperiment: SummarizedExperiment container (2020).
16. K. Blighe, S. Rana, M. Lewis, EnhancedVolcano: Publication-ready volcano plots with enhanced colouring and labeling (2020).
17. S. Durinck, et al., BioMart and Bioconductor: a powerful link between biological databases and microarray data analysis. Bioinformatics 21, 3439–3440 (2005).
18. S. Durinck, P. T. Spellman, E. Birney, W. Huber, Mapping identifiers for the integration of genomic datasets with the R/Bioconductor package biomaRt. Nat. Protoc. 4, 1184–1191 (2009).
19. A. Alexa, J. Rahnenfuhrer, topGO: Enrichment Analysis for Gene Ontology (2020).
20. V. Tams, J. H. Nickel, A. Ehring, M. Cordellier, Insights into the genetic basis of predator-induced response in Daphnia galeata. Ecol. Evol. 10, 13095–13108 (2020).
21. A. Oliver, et al., Phenotypic and transcriptional response of *Daphnia pulicaria* to the combined effects of temperature and predation. PLoS One 17, e0265103 (2022).
22. O. Tills, et al., Transcriptomic responses to predator kairomones in embryos of the aquatic snail *Radix balthica*. Ecol. Evol. 8, 11071–11082 (2018).
23. M. Kanehisa, S. Goto, KEGG: kyoto encyclopedia of genes and genomes. Nucleic Acids Res. 28, 27–30 (2000).

24. S. Falcon, R. Gentleman, Using GOstats to test gene lists for GO term association. Bioinformatics 23, 257–258 (2007).
25. G. Yu, L.-G. Wang, Y. Han, Q.-Y. He, clusterProfiler: an R package for comparing biological themes among gene clusters. OMICS: A Journal of Integrative Biology 16, 284–287 (2012).
26. G. Yu, Q.-Y. He, ReactomePA: an R/Bioconductor package for reactome pathway analysis and visualization. Molecular BioSystems 12, 477–479 (2016).
27. L. Ruzicka, et al., The Zebrafish Information Network: new support for non-coding genes, richer Gene Ontology annotations and the Alliance of Genome Resources. Nucleic Acids Res. 47, D867–D873 (2019).
28. R. Elkon, et al., RFX transcription factors are essential for hearing in mice. Nat. Commun. 6, 8549 (2015).
29. L. Desban, et al., Lateral line hair cells integrate mechanical and chemical cues to orient navigation. bioRxiv, 2022.08.31.505989 (2022).
30. C. A. Smith, E. J. Want, G. O'Maille, R. Abagyan, G. Siuzdak, XCMS: processing mass spectrometry data for metabolite profiling using nonlinear peak alignment, matching, and identification. Anal. Chem. 78, 779–787 (2006).
31. C. Kuhl, R. Tautenhahn, C. Böttcher, T. R. Larson, S. Neumann, CAMERA: an integrated strategy for compound spectra extraction and annotation of liquid chromatography/mass spectrometry data sets. Anal. Chem. 84, 283–289 (2012).
32. R. Guha, Chemical Informatics Functionality in R. J. Stat. Softw. 18 (2007).
33. A. Voicu, N. Duteanu, M. Voicu, D. Vlad, V. Dumitrascu, The rcdk and cluster R packages applied to drug candidate selection. J. Cheminform. 12, 3 (2020).
34. Z. Pang, et al., MetaboAnalyst 5.0: narrowing the gap between raw spectra and functional insights. Nucleic Acids Res. (2021) https:/doi.org/10.1093/nar/gkab382.
35. S. Li, et al., Constructing a fish metabolic network model. Genome Biol. 11, R115 (2010).
36. K. O'Shea, et al., DIMEdb: an integrated database and web service for metabolite identification in direct infusion mass spectrometery. bioRxiv, 291799 (2018).
37. T. Kind, et al., LipidBlast in silico tandem mass spectrometry database for lipid identification. Nat. Methods 10, 755–758 (2013).
38. UniProt Consortium, UniProt: a worldwide hub of protein knowledge. Nucleic Acids Res. 47, D506–D515 (2019).
39. Y. Djoumbou Feunang, et al., ClassyFire: automated chemical classification with a comprehensive, computable taxonomy. J. Cheminform. 8, 61 (2016).
40. Z. Pang, J. Chong, S. Li, J. Xia, MetaboAnalystR 3.0: Toward an Optimized Workflow for Global Metabolomics. Metabolites 10 (2020).
41. Z. Pang, et al., Using MetaboAnalyst 5.0 for LC-HRMS spectra processing, multi-omics integration and covariate adjustment of global metabolomics data. Nat. Protoc. (2022) https:/doi.org/10.1038/s41596-022-00710-w.
42. D. Szklarczyk, et al., STITCH 5: augmenting protein-chemical interaction networks with tissue and affinity data. Nucleic Acids Res. 44, D380–4 (2016).
43. N. T. Doncheva, J. H. Morris, J. Gorodkin, L. J. Jensen, Cytoscape StringApp: Network Analysis and Visualization of Proteomics Data. J. Proteome Res. 18, 623–632 (2019).
44. P. Shannon, et al., Cytoscape: a software environment for integrated models of biomolecular interaction networks. Genome Res. 13, 2498–2504 (2003).
45. K. J. Livak, T. D. Schmittgen, Analysis of relative gene expression data using real-time quantitative PCR and the 2- ΔΔCT method. Methods 25, 402–408 (2001).

46. C. A. Schneider, W. S. Rasband, K. W. Eliceiri, NIH Image to ImageJ: 25 years of image analysis. Nat. Methods 9, 671–675 (2012).
47. J. Charmant, Contributors, Kinovea (0.9.5) (2021).
48. RStudio Team, RStudio: Integrated Development Environment for R (2020).
49. H. Wickham, ggplot2: Elegant Graphics for Data Analysis (2016).
50. L. Komsta, outliers: Tests for outliers (2011).
51. M. J. Mazerolle, AICcmodavg: Model selection and multimodel inference based on (Q)AIC(c) (2020).
52. W. N. Venables, B. D. Ripley, Modern Applied Statistics with S (2002).
53. J. Oksanen, et al., vegan: Community Ecology Package (2020).
54. V. Q. Vu, ggbiplot: A ggplot2 based biplot (2011).
55. A. Zeileis, T. Hothorn, Diagnostic Checking in Regression Relationships. R News 2, 7–10 (2002).
56. S. Mangiafico, rcompanion: Functions to support extension education program evaluation. R package version 1 (2018).
57. M. L. Delignette-Muller, C. Dutang, fitdistrplus: An R Package for Fitting Distributions. Journal of Statistical Software 64, 1–34 (2015).
58. A. W. Bowman, A. Azzalini, R package sm: nonparametric smoothing methods (version 2.2-5.7) (2021).
59. P. Martinez Arbizu, pairwiseAdonis: Pairwise Multilevel Comparison using Adonis (2017).
60. J. Cohen, Statistical power analysis for the behavioural sciences (2nd edn.), Hillside, NJ: Erlbaum (NJ: Erlbaum, 1988).
61. M. S. Ben-Shachar, D. Lüdecke, D. Makowski, effectsize: Estimation of Effect Size Indices and Standardized Parameters. Journal of Open Source Software 5, 2815 (2020).
62. S. S. Sawilowsky, New Effect Size Rules of Thumb. J. Mod. Appl. Stat. Methods 8, 26 (2009).
63. C. B. Kimmel, W. W. Ballard, S. R. Kimmel, B. Ullmann, T. F. Schilling, Stages of embryonic development of the zebrafish. Dev. Dyn. 203, 253–310 (1995).
64. L. J. G. Barcellos, G. L. Volpato, R. E. Barreto, I. Coldebella, D. Ferreira, Chemical communication of handling stress in fish. Physiol. Behav. 103, 372–375 (2011).
65. J. Tokarz, W. Norton, G. Möller, M. Hrabé de Angelis, J. Adamski, Zebrafish 20β-hydroxysteroid dehydrogenase type 2 is important for glucocorticoid catabolism in stress response. PLoS One 8, e54851 (2013).
66. M. R. Crossland, A. A. Salim, R. J. Capon, R. Shine, The Effects of Conspecific Alarm Cues on Larval Cane Toads (*Rhinella marina*). J. Chem. Ecol. 45, 838–848 (2019).
67. L. C. Weiss, et al., Identification of Chaoborus kairomone chemicals that induce defences in Daphnia. Nat. Chem. Biol. 14, 1133–1139 (2018).
68. R. Saroya, R. Smith, C. Seymour, C. Mothersill, Injection of resperpine into zebrafish, prevents fish to fish communication of radiation-induced bystander signals: confirmation in vivo of a role for serotonin in the mechanism. Dose Response 8, 317–330 (2009).
69. K. V. Parra, J. C. Adrian Jr, R. Gerlai, The synthetic substance hypoxanthine 3-N-oxide elicits alarm reactions in zebrafish (*Danio rerio*). Behav. Brain Res. 205, 336–341 (2009).
70. A. S. Mathuru, et al., Chondroitin fragments are odorants that trigger fear behavior in fish. Curr. Biol. 22, 538–544 (2012).
71. K. B. Døving, S. Lastein, The alarm reaction in fishes--odorants, modulations of responses, neural pathways. Ann. N. Y. Acad. Sci. 1170, 413–423 (2009).
72. F. Kermen, et al., Stimulus-specific behavioral responses of zebrafish to a large range of odors exhibit individual variability. BMC Biol. 18, 66 (2020).

73. J. Canzian, B. D. Fontana, V. A. Quadros, D. B. Rosemberg, Conspecific alarm substance differently alters group behavior of zebrafish populations: Putative involvement of cholinergic and purinergic signaling in anxiety- and fear-like responses. Behav. Brain Res. 320, 255–263 (2017).
74. G. E. Brown, J. C. Adrian, E. Smyth, H. Leet, S. Brennan, Ostariophysan Alarm Pheromones: Laboratory and Field Tests of the Functional Significance of Nitrogen Oxides. J. Chem. Ecol. 26, 139–154 (2000).
75. Y. Ito, T. Suzuki, T. Shirai, T. Hirano, Presence of cyclic betaines in fish. Comparative Biochemistry and Physiology Part B: Comparative Biochemistry 109, 115–124 (1994).
76. R. X. Poulin, et al., Chemical encoding of risk perception and predator detection among estuarine invertebrates. Proceedings of the National Academy of Sciences 115, 662–667 (2018).
77. Y. Nakagawa, H. Sonobe, "Subchapter 98A - 20-Hydroxyecdysone" in Handbook of Hormones, Y. Takei, H. Ando, K. Tsutsui, Eds. (Academic Press, 2016), pp. 560–e98A–2.
